# Disruption of the Nav1.8/Ankyrin-3 interaction in nociceptors reduces pathological pain-like behaviors in mice

**DOI:** 10.1016/j.xcrm.2026.103090

**Published:** 2026-10-06

**Authors:** Rasheen Powell, Xiangsunze Zeng, Bruna Lenfers Turnes, Hyoung Woo Kim, Veselina Petrova, Selwyn S. Jayakar, Emily Shea, Keunjung Heo, Emma Cropper, Maryam Arab, Georgios Kimourtzis, Juan A. Oses-Prieto, Alma L. Burlingame, Stephen G. Waxman, Sulayman D. Dib-Hajj, Clifford J. Woolf

**Affiliations:** 1 F.M. Kirby Neurobiology Center, Boston Children’s Hospital, Boston, MA, USA; 2 Department of Neurobiology, Harvard Medical School, Boston, MA, USA; 3 Department of Pharmaceutical Chemistry, University of California, San Francisco, San Francisco, CA, USA; 4 Department of Neurology, Yale School of Medicine, New Haven, CT, USA

**Keywords:** pain, neuropathic pain, Nav1.8 sodium channel, ankyrin, nociceptor, lipidated peptide

## Abstract

Treating chronic neuropathic pain is challenging due to its complex pathophysiology. Persistent changes in the somatosensory system prolong heightened states of stimulus-evoked and spontaneous pain. Nociceptor dorsal root ganglion (DRG) neurons are key contributors to neuropathic pain due to pathological plasticity following injury, leading to spontaneous ectopic and stimulus-evoked firing. Preclinical strategies to inhibit Nav1.8, a nociceptor-specific voltage-gated sodium channel, are successful in reducing neuropathic pain but have yet to produce similar results in clinical settings. Here, we evaluate the pharmacotherapeutic potential of the Nav1.8/Ank3 interaction in neuropathic pain, utilizing electrophysiology, biochemistry, and proteomic approaches. We report that Nav1.8/Ank3 interactions are necessary for maintaining pathological nociceptor hyperexcitability in neuroma pain and that inhibition of this interaction with a short lipidated peptide (SLiP) is sufficient to alleviate neuropathic pain-like behaviors in mice. This work highlights the value of reevaluating targets for pharmacological intervention to produce analgesia specific to pathological states.

## Highlights

• Nav1.8/Ank3 interaction can be disrupted *in vivo* using a short lipidated peptide
• Disrupting Nav1.8/Ank3 binding reduces pathological pain-like behaviors in mice
• Nav1.8/Ank3 peptide spares acute protective nociception in uninjured animals
• Nav1.8/Ank3 peptide specifically reduces Nav1.8 conductance in sensitized neurons

## Graphical abstract

**Figure:**
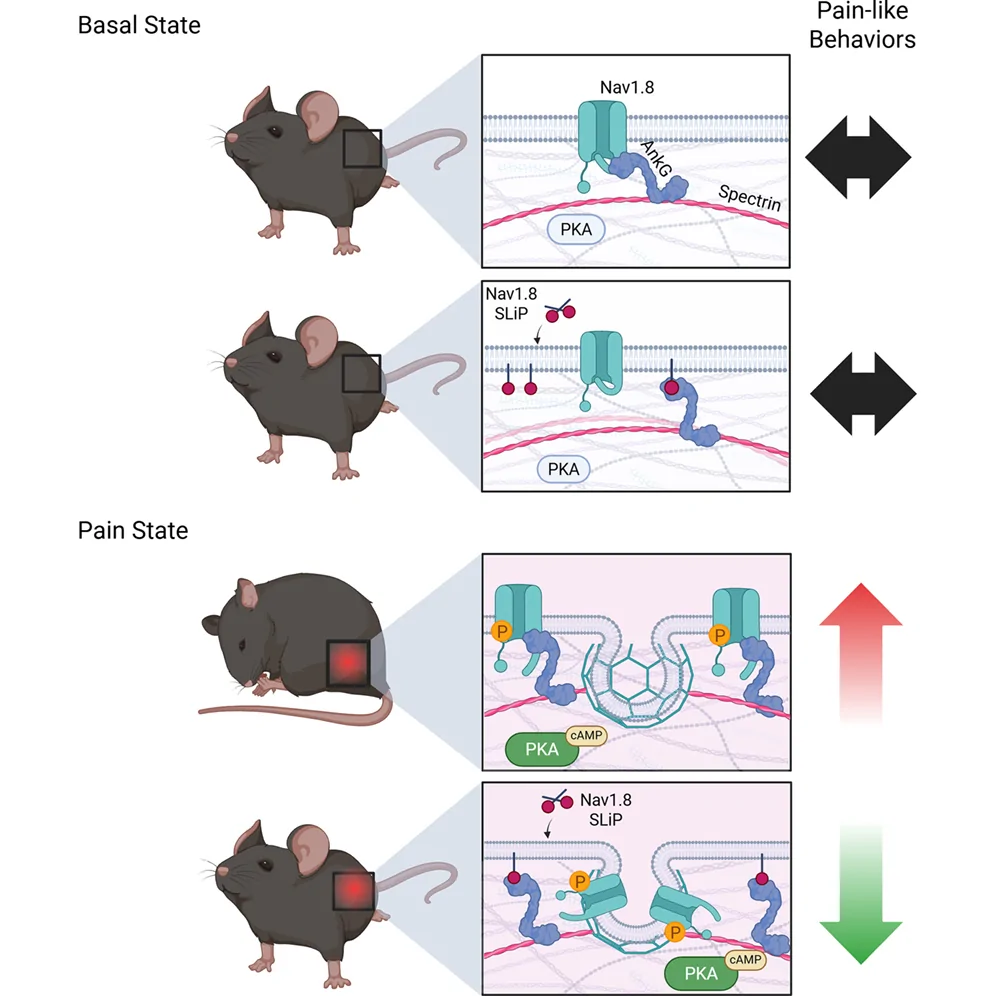

## Teaser

Chronic pain is a complex neurobiological problem whose solution is elusive. Powell et al. describe an approach to reduce chronic neuropathic pain in rodent models using a short lipidated peptide designed to inhibit the interaction between Nav1.8 and Ankyrin-3.

## Introduction

Acute pain is a somatosensory experience that promotes organism survival through avoidance of potentially harmful stimuli^1^^,^^2^; however, chronification of pain results in a debilitating pathological state.^3^^,^^4^ Neuropathic pain, resulting from direct or indirect insults to neurons, arises from pathological neurocircuitry changes in peripheral and central neurons. These changes drive bouts of spontaneous pain, paresthesia, and both stimulus-evoked hyperalgesia and allodynia.^5^^,^^6^ An ever-growing body of work has highlighted the intrinsic and extrinsic factors that contribute to the complexity of chronic neuropathic pain.^7^^,^^8^^,^^9^^,^^10^^,^^11^^,^^12^ Current treatment strategies for chronic neuropathic pain include myriad pharmacological and non-pharmacological approaches^13^^,^^14^^,^^15^^,^^16^^,^^17^^,^^18^; however, there is a need for more efficacious analgesics with limited adverse effect profiles. In consequence, there have been significant efforts to identify the key drivers of pathological pain that can be pharmacologically modulated to produce analgesia.

Voltage-gated sodium channels (Navs) are attractive targets for analgesic development due to their electrogenic function in neurons. Nociceptors preferentially express a subset of these channels—Nav1.7, Nav1.8, and Nav1.9^19^^,^^20^^,^^21^—and their role in promoting nociception has been validated clinically and preclinically. Nav1.8 is particularly noteworthy for its large contribution to the action potential upstroke in nociceptors.^22^^,^^23^^,^^24^^,^^25^^,^^26^^,^^27^^,^^28^ Genetic and pharmacological manipulation of Nav1.8 produces analgesic-like effects in preclinical models of neuropathic pain^25^^,^^29^^,^^30^; however, clinical validation of these observations has yet to be achieved. Recently, the Food and Drug Administration (FDA) approved a Nav1.8 selective inhibitor, suzetrigine, for acute pain, validating Nav1.8 as a target for treating acute inflammatory pain states.^12^^,^^15^^,^^31^^,^^32^ However, its efficacy in chronic pain conditions is still being investigated.

Nav channels interact with multiple intracellular proteins that govern their membrane expression and gating.^24^^,^^33^^,^^34^^,^^35^^,^^36^ Nav1.8 is particularly sensitive to auxiliary protein interactors; it has an idiosyncratic PKA phosphorylation motif that, when phosphorylated, increases Nav1.8 current density through forward trafficking to the membrane.^23^^,^^37^^,^^38^ Nav1.8 also binds to Magi-1, a scaffold protein that protects it from Nedd4-2-mediated degradation, and disruption of this interaction decreases Nav1.8 membrane expression, reducing pain-like behaviors in mice.^24^^,^^26^ These findings illustrate the reliance Nav1.8 has on intracellular binding partners to contribute to nociceptor excitability.

Among these binding partners are ankyrins—a family of membrane adaptor proteins that link membrane-bound proteins to intracellular cytoskeletal elements. Ankyrins are expressed across multiple cell types where they organize proteins into subcellular domains.^39^^,^^40^^,^^41^^,^^42^^,^^43^ The three ankyrin isoforms (Ank1, Ank2, and Ank3) share similarities in their overall structure: a membrane-binding domain, a spectrin-binding domain, a death domain, and a disorganized C-terminal domain. Curiously, Ank2 and Ank3 have giant isoforms (>400 kDa) that function as molecular hubs for protein-protein interactions in electrically excitable cells.^44^^,^^45^^,^^46^^,^^47^^,^^48^^,^^49^ Nav channels have a *bona fide* ankyrin-binding domain (ABD) within their intracellular loop 2 region that has been extensively studied in various tissues.^33^^,^^39^^,^^43^^,^^50^ Any loss of Nav/Ank interactions disrupt proper localization of Nav channels impairing tissue function.^44^^,^^51^^,^^52^ Strikingly, Nav1.8’s ABD was found to constitutively interact with ankyrins and displace other Nav ABDs at axon initial segments (AISs).^53^ This suggests that the Nav1.8/Ank interaction in nociceptors may be differentiated from Nav/Ank interactions in other cells. However, there has been limited characterization of Nav1.8/Ank interactions in the context of pain.

In this study, we aimed to investigate the Nav1.8/Ank interaction in nociceptors and evaluate its potential as a target for pharmacological intervention in neuropathic pain. We present evidence that the Nav1.8/Ank interaction is a critical component of pathological but not acute physiological nociception. Our findings also provide functional data that the Nav1.8 and Ank3 interaction is crucial for persistent hyperexcitability after peripheral nerve injury. Pharmacological interruption of this interaction produces analgesia in models of neuropathic and acute inflammatory pain. Overall, our data uncovers a mechanism that maintains nociceptor hyperexcitability during pathological but not physiological pain states, providing a framework for the development of effective non-opioid analgesics that do not disrupt the protective function of nociceptive pain.

## Results

### Ankyrin isoforms display distinct expression patterns in peripheral sensory neurons

Ankyrins are a family of ubiquitously expressed scaffold adaptor proteins that localize membrane bound proteins to distinct subcellular domains. The three ankyrin isoforms (Ank1, Ank2, and Ank3) share a similar overall structure (Figure 1A). Extensive work has characterized the interaction between Nav channels and ankyrins,^43^^,^^50^^,^^53^ uncovering a conserved ABD in all Nav channel isoforms. One characteristic of this motif is that the Nav1.8 ABD is highly conserved between species (Figure 1B), emphasizing the importance of this interaction.

**Figure 1:**
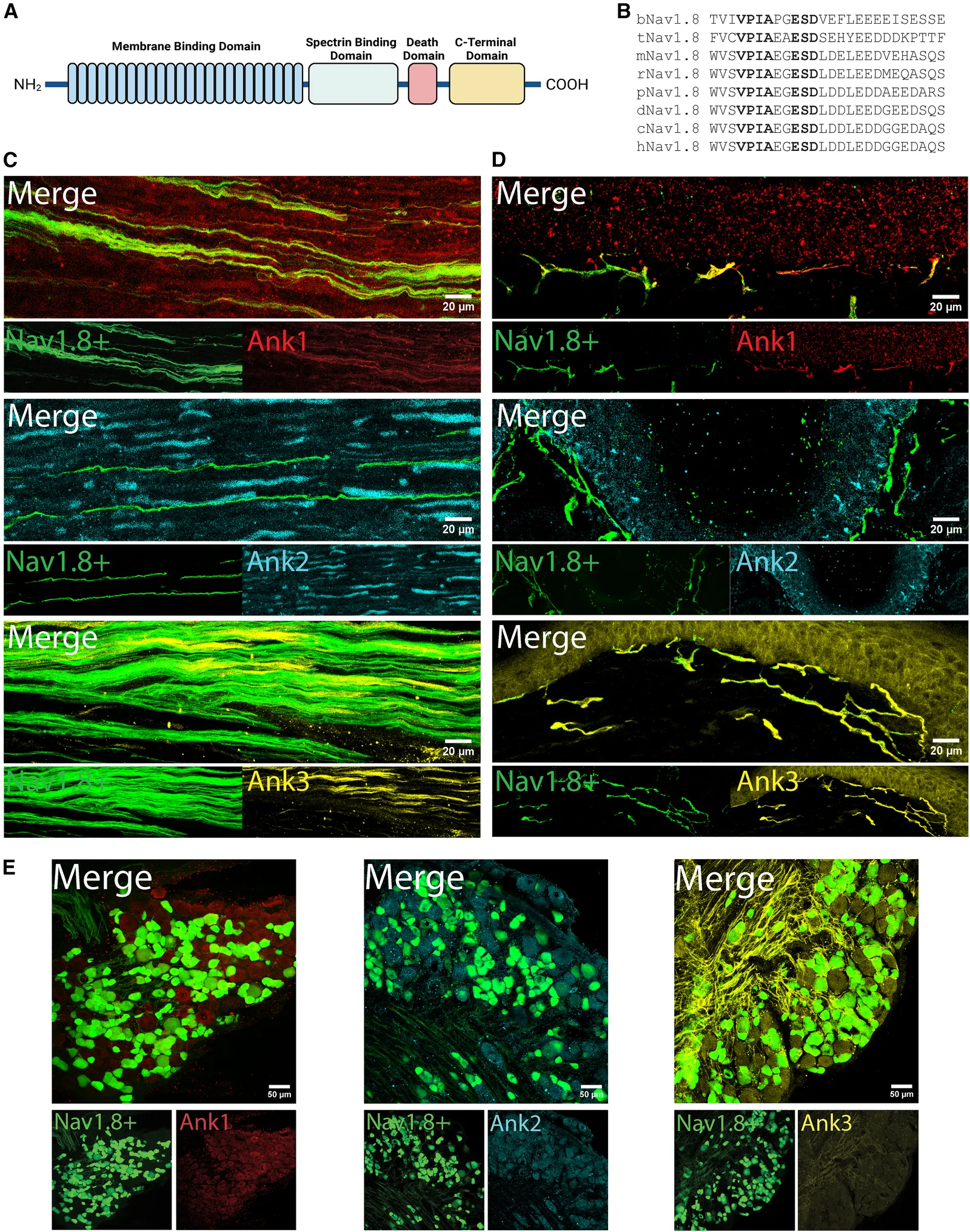
Ankyrins display distinct distribution patterns in Nav1.8^Tdtomato^ neurons (A) Schematic of canonical ankyrin domains. (B) Sequence alignment of verified and putative Nav1.8 ankyrin-binding domains (bolded) across various species: *Dicentrarchus labrax* (bNav1.8), *Terrapene triunguis* (tNav1.8), *Mus Musculus* (mNav1.8), *Rattus norvegicus* (rNav1.8), *Sus scrofa* (pNav1.8), *Canis familiaris* (dNav1.8), *Pan Troglodytes* (cNav1.8), and *Homo sapiens* (hNav1.8). Sequences were accessed from UniProt.org and aligned using its online sequence alignment tool. Sequences were accessed from both Swiss-Prot and TrEMBL databases. (C–E) Representative immunohistochemical images of Ank1, Ank2, and Ank3 expression in Nav1.8^Tdtomato^ (green) expressing sensory neurons in different anatomical compartments. (C) Sciatic nerve axons. Scale bars are 20 μm in length.(D) Glabrous skin of the hind paw. Scale bars are 20 μm in length. (E) Dorsal root ganglion. Scale bars are 50 μm in length.

Using immunofluorescent staining, we visualized the expression of the three ankyrin isoforms in different peripheral compartments. Since peripheral sensory neurons are heterogenous, we used a Nav1.8^Tdtomato^/*Thy1*^YFP^ reporter mouse to broadly label two sensory neuron subpopulations: small/medium (Nav1.8^+^) and large (*Thy1*^+^) diameter neurons. We observed all three ankyrin isoforms in sensory neuron axons, with each displaying a distinct expression profile (Figure 1C). Immunoreactivity for Ank1 and Ank3 was observed in Nav1.8+ axons (Figure 1C, top and bottom), while immunoreactivity for Ank2 was not detected in Nav1.8+ axons (Figure 1C, middle). A similar immunoreactivity pattern was observed in the glabrous skin of the hind paw (Figure 1D). When evaluating expression in the soma, Ank1 and Ank2 were found expressed in both Nav1.8+ and Nav1.8− neurons, while Ank3 immunoreactivity was weaker in comparison to the axon or axon terminals (Figure 1E). These data suggest that the different ankyrin isoforms may have fiber-compartment-specific roles.

Additionally, we utilized the Pain-Seq^54^ and Sensoryomics^55^ platforms to understand the transcriptomic profile of ankyrin isoforms across defined sensory neuron subtypes. We found that, in mice, predominantly non-nociceptive cell types (i.e., *Ntrk3*-high and *Pvalb*) did not express Ank3 at high levels, while Ank3 was highly expressed across small-diameter sensory neuron subtypes (i.e., Atf3, *Calca*+*Sstr2*, and *Sst*) (Figure S1A). In humans, Ank1 is highly expressed in Aβ fibers, whereas Ank2 and Ank3 show preferential expression in nociceptors (i.e., “cold nociceptors,” *PENK1*+; Figures S1B and S1C). These data reveal that Ank3 is found in sensory neuron subpopulations associated with pain in mice and humans at both a transcriptomic and protein level.

### Knockdown of Ank3 reduces TTX-R Nav channel conductance in cultured eDRG neurons

Experiments conducted by Montersino et al.^53^ revealed that Nav1.8’s ABD constitutively bound Ank2 and Ank3 with high affinity.^53^ Therefore, we aimed to determine which ankyrin isoform promotes Nav1.8 conductance in sensory neurons using a viral knockdown approach in an established eDRG neuron culture system.^56^^,^^57^

First, we confirmed the expression of the three ankyrin isoforms and Nav1.8 in cultured eDRG neurons (Figure 2A). Then, we utilized virally delivered short hairpin RNA (shRNA) to knockdown each ankyrin isoform. Immunofluorescent staining revealed that targeted shRNAs produced a partial knockdown of the target ankyrin isoforms (Figure 2C; Figures S2A–S2C) when compared to the empty vector control (Figure 2B). These results were verified by qPCR, which demonstrated a robust reduction in target ankyrin mRNA (Figure 2D).

**Figure 2:**
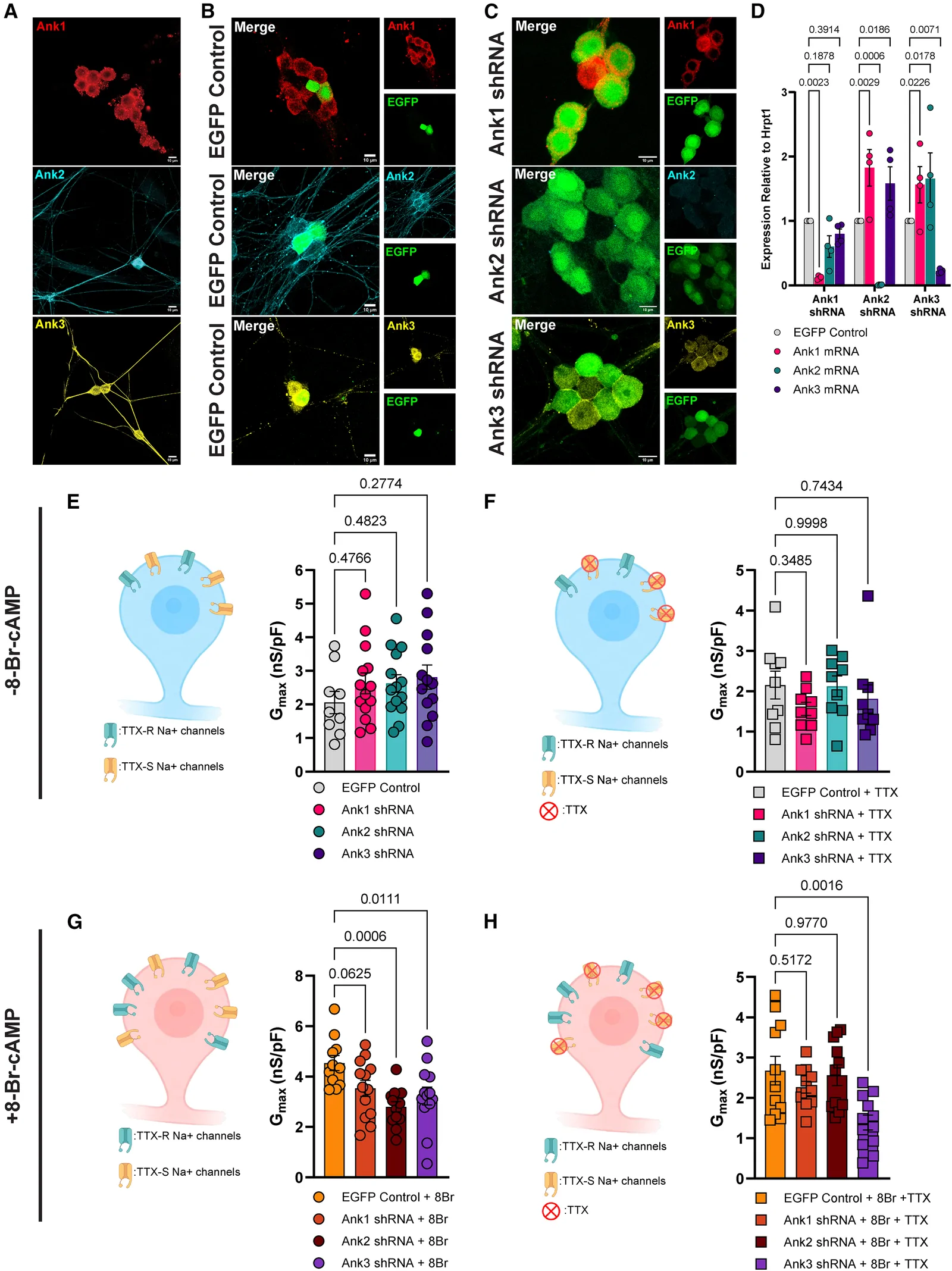
shRNA-mediated knockdown of Ank3 reduces TTX-R sodium currents in cultured eDRG neurons only in the presence of cAMP kinase activity (A) Representative immunofluorescent images displaying expression of: (top) Ank1, (middle) Ank2, and (bottom) Ank3 in cultured eDRG neurons. (B) Representative immunofluorescent images of eDRG neurons transduced with an EGFP control adeno-associated virus (AAV). (C) Representative immunofluorescent images displaying expression of ankyrin isoforms following AAV-mediated shRNA knockdown of targeted ankyrin isoform in cultured eDRG neurons: Ank1 (top), Ank2 (middle), and Ank3 (bottom). Successfully transduced neurons express EGFP (green). (A–C) Scale bars are 10 μm in length. (D) Quantification of Ank mRNA transcripts following AAV shRNA-mediated knockdown. Bars represent the average mRNA expression relative to *Hprt1* expression (*n* = 4 independent experiments). (E) Maximum Nav channel conductance following shRNA knockdown of ankyrin isoforms: EGFP control group (*n* = 9), Ank1 (*n* = 14), Ank2 (*n* = 14), and Ank3 (*n* = 13). (F) Maximum conductance of TTX-R Nav channels following shRNA knockdown of ankyrins in the presence of TTX: EGFP control (*n* = 9), Ank1 (*n* = 9), Ank2 (*n* = 9), and Ank3 (*n* = 9). (G) Maximum conductance of total Nav channels following shRNA knockdown in the presence of 8-Br-cAMP: EGFP control (*n* = 11), Ank1 (*n* = 13), Ank2 (*n* = 12), and Ank3 (*n* = 13). (H) Maximum conductance of TTX-R Nav channels following shRNA knockdown in the presence of 8-Br-cAMP and TTX: EGFP control (*n* = 11), Ank1 (*n* = 11), Ank2 (*n* = 11), and Ank3 (*n* = 12). For (E)–(H), data represent pooled replicates from three independent experiments. Statistical significance was determined using one-way ANOVA with multiple comparisons and Dunnett post-hoc correction. All data presented as mean ± SEM. Illustrative graphics were created in BioRender. Powell, R. (2026) https://BioRender.com/hmxwwa5.

Next, we evaluated the electrophysiological consequence of knocking down each ankyrin isoform by quantifying the maximal Nav channel conductance (G_max_) under various conditions. Knockdown of each ankyrin isoform had no significant effect on G_max_ when compared to the EGFP control (Figure 2E; EGFP control; 2.1 ± 0.3 nS/pF, *Ank1* shRNA; 2.6 ± 0.3 nS/pF, *Ank2* shRNA; 2.6 ± 0.3 nS/pF, *Ank3* shRNA; 2.8 ± 0.4 nS/pF). We repeated these recordings in the presence of TTX to block TTX-sensitive (TTX-S) sodium channel isoforms (Nav1.1, 1.2, 1.3, 1.6, and 1.7) and isolate Nav1.8 (TTX-R) currents. Under these conditions, there was no significant difference in G_max_ (Figure 2F) between all the groups (EGFP control; 2.2 ± 0.4 nS/pF, *Ank1* shRNA; 1.6 ± 0.2 nS/pF, *Ank2* shRNA; 2.1 ± 0.3 nS/pF, *Ank3* shRNA; 1.8 ± 0.4 nS/pF). These data suggest that loss of any of the three ankyrin isoforms has no significant effect on sodium channel conductance in sensory neurons in a physiological state.

During pathological pain, various signaling pathways promote nociceptor hyperexcitability. One pathway acts through cAMP-dependent kinases, like PKA, to increase Nav1.8 membrane expression^23^^,^^37^^,^^58^ driving sensory neuron hyperexcitability (Figure S3). Therefore, we repeated G_max_ recordings in the presence of the cAMP kinase activator 8-Br-cAMP (Figures 2G and 2H), to partially recapitulate pathological signaling. In this condition, there was a significant decrease in the total G_max_ (Figure 2G) for all shRNA knockdown groups compared to control (Figure 2G: EGFP control; 4.5 ± 0.3 nS/pF, *Ank1* shRNA; 3.5 ± 0.3 nS/pF, *Ank2* shRNA; 2.8 ± 0.2 nS/pF, *Ank3* shRNA; 3.2 ± 0.4 nS/pF), suggesting that all ankyrin isoforms can play a role promoting Nav channel conductance in pathological conditions, which aligns with previous reports.^33^^,^^43^^,^^59^^,^^60^^,^^61^ Again, we isolated TTX-R sodium currents in the presence of 8-Br-cAMP, which revealed that Ank3 knockdown resulted in a significant reduction in maximum TTX-R G_max_ (Figure 2H: EGFP control; 2.7 ± 0.4 nS/pF, *Ank1* shRNA; 2.3 ± 0.2 nS/pF, *Ank2* shRNA; 2.6 ± 0.3 nS/pF, *Ank3* shRNA; 1.4 ± 0.2 nS/pF). These data suggest that Nav1.8 conductance is sensitive to Ank3 expression levels in cAMP-kinase-stimulated conditions.

### A short lipidated peptide mimicking the Nav1.8 ABD reduces Nav1.8 current in a cAMP-kinase-dependent manner

To investigate the pharmacological potential of the Nav1.8/Ank3 interaction, we employed a short lipidated peptide (SLiP) approach. Ion-channel-targeted SLiPs have been shown to modulate neuronal activity and function.^24^^,^^26^^,^^56^^,^^62^^,^^63^ Therefore, we designed a SLiP based on the Nav1.8 ABD and used it to characterize the electrophysiological consequences of disrupting Nav1.8 and Ank3 interactions.

Previous work has revealed that Nav channels preferentially bind to two different sites on ankyrins^64^; however, there is no evidence describing whether different Nav channels have different affinities for each site. To explore if Nav channels occupy different sites on ankyrins, we utilized Boltz-2, accessed through Neurosnap,^65^ to predict where the Nav1.8 ABD would bind the membrane-binding domains of Ank1, Ank2, or Ank3 (Figure 3A) in comparison to the Nav1.6 and Nav1.7 ABD. Boltz-2 modeling predicted that the Nav1.8 ABD would not overlap with Nav 1.6 or 1.7 ABDs, supporting previous data suggesting that Nav channels may bind to different sites on ankyrins.^66^

**Figure 3:**
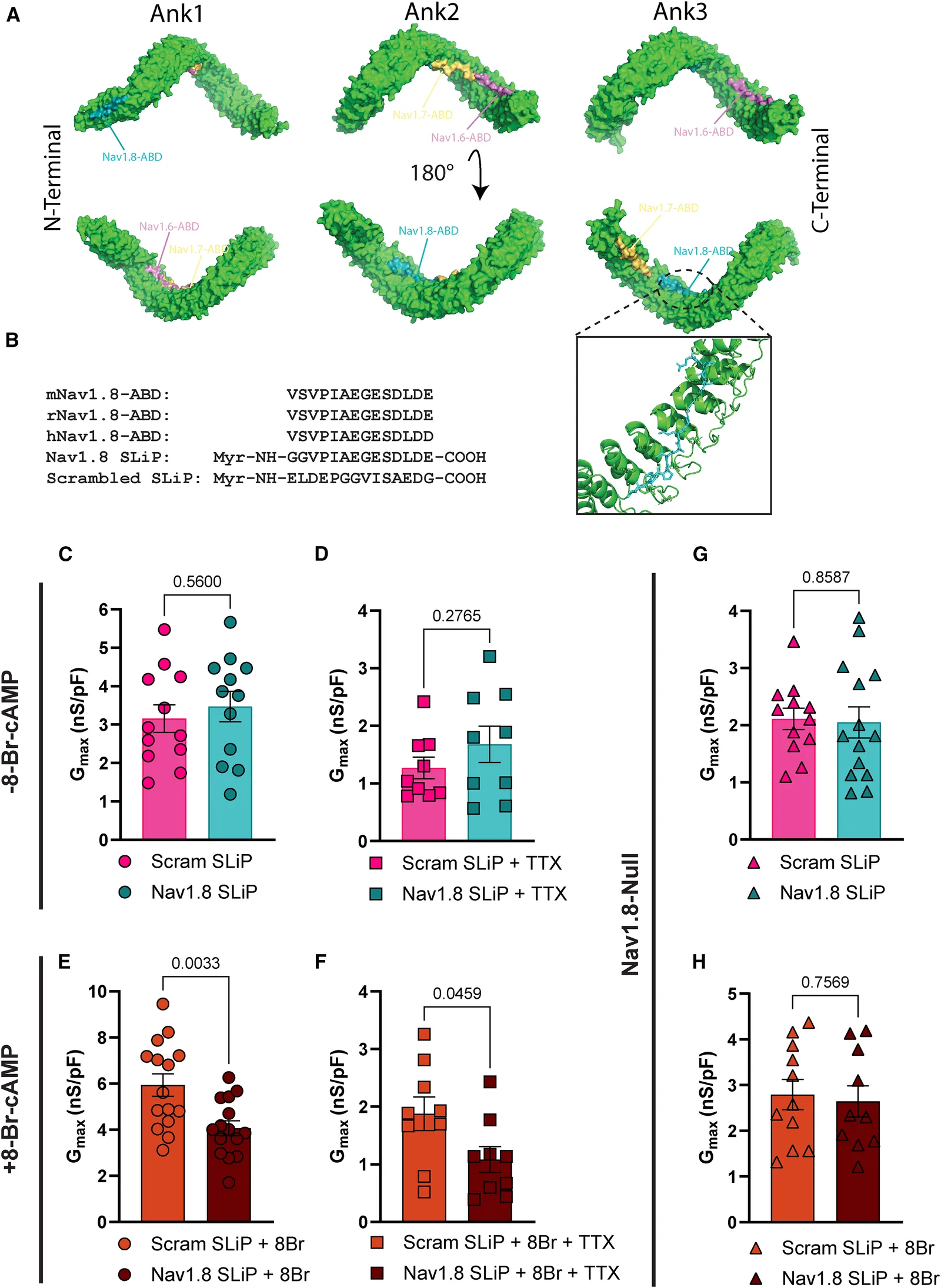
A SLiP mimicking the Nav1.8 ABD reduces TTX-R current in cultured eDRG neurons only in the presence of cAMP-kinase-induced activity (A) (Top) Representative 3D structures predicting potential binding sites for Nav1.8 ABD (cyan), Nav1.6 ABD (magenta), and Nav1.7 ABD (yellow) on Ank1 (left), Ank2 (middle), and Ank3 (right). Structures generated using Boltz-2, accessed through the Neurosnap.ai interface, showing the predicted binding sites of Nav1.8 ABD and Nav1.6 ABD or Nav1.7 ABD on ankyrin isoforms. (Bottom) 3D structures rotated 180° about their central axis. (B) Sequence alignment of peptides used and Nav1.8 sequence in *Mus musculus*, *Rattus norvegicus*, and *Homo sapiens*. (C) Maximum Nav channel conductance following incubation with either the Scram (*n* = 12) or Nav1.8 SLiP (*n* = 12). (D) Maximum conductance of TTX-R Nav channels following incubation with either the Scram (*n* = 9) or Nav1.8 SLiP (*n* = 9). (E) Maximum Nav channel conductance following incubation with either the Scram (*n* = 15) or Nav1.8 SLiP (*n* = 15) in the presence of 8-Br-cAMP. (F) Maximum conductance of TTX-R Nav channels following incubation with either the Scram (*n* = 9) or Nav1.8 SLiP (*n* = 9) in the presence of 8-Br-cAMP. (G) Maximum Nav channel conductance in aDRG Nav1.8-null neurons following incubation with either the Scram (*n* = 12) or Nav1.8 SLiP (*n* = 14). (H) Maximum Nav channel conductance in aDRG Nav1.8-null neurons following incubation with either the Scram (*n* = 11) or Nav1.8 SLiP (*n* = 10) in the presence of 8-Br-cAMP. For (C)–(H), data represent pooled replicates from three independent experiments. Statistical significance determined using two-tailed unpaired Student’s *t* test. All data presented as mean ± SEM.

Next, we designed two SLiPs: one based on the Nav1.8 ABD (Nav1.8 SLiP) and a scrambled sequence control (Scram SLiP; Figure 3B). We confirmed that the Nav1.8 SLiP disrupted Nav1.8 and Ank3 binding through a co-immunoprecipitation assay (Figure S4). Then we measured G_max_ in neurons that were exposed to either SLiP, but there was no significant difference in G_max_ between groups (Figure 3C; Scram SLiP 3.2 ± 0.4 nS/pF and Nav1.8 SLiP 3.5 ± 0.4 nS/pF). A similar trend was observed when isolating TTX-R currents (Figure 3D; Scram SLiP 1.3 ± 0.2 nS/pF and Nav1.8 SLiP 1.7 ± 0.3 nS/pF). However, pre-treatment with 8-br-cAMP produced a significant reduction in total G_max_ (Figure 3E; Scram SLiP 5.9 ± 0.5 nS/pF and Nav1.8 SLiP 4.1 ± 0.3 nS/pF) and TTX-R G_max_ (Figure 3F; Scram SLiP 1.9 ± 0.3 nS/pF and Nav1.8 SLiP 1.1 ± 0.2 nS/pF).

We then endeavored to confirm the specificity of the Nav1.8 SLiP. To do this, we performed whole-cell patch-clamp recordings on cultured adult DRG neurons from Nav1.8-null mice. The Nav1.8 SLiP had no effect on G_max_ in the basal state (Figure 3G; Scram SLiP 2.1 ± 0.2 nS/pF and Nav1.8 SLiP 2.0 ± 0.3 nS/pF) and addition of 8-Br-cAMP failed to produce a reduction in G_max_ (Figure 3H; Scram SLiP 2.8 ± 0.3 nS/pF and Nav1.8 SLiP 2.6 ± 0.3 nS/pF), providing evidence that the Nav1.8 SLiP selectively displaces Nav1.8 from ankyrins. This was further confirmed through live cell fluorescent imaging of eDRG neurons transfected with halo-tagged Nav1.7 and Nav1.8 constructs (Figure S5). The Nav1.8 SLiP caused a decrease in the Nav1.8-halo fluorescent signal following addition of 8-Br-cAMP, without affecting the Nav1.7-halo fluorescent signal. Overall, these data provide evidence that the Nav1.8 SLiP selectively reduces Nav1.8 channel conductance in sensory neurons in PKA-activated conditions.

### Nav1.8 SLiP reduces electrophysiological metrics associated with hyperexcitability

The above data show the efficacy of the Nav1.8 SLiP in decreasing total and TTX-R G_max_ in PKA-activated conditions. Next, we investigated whether this reduction in Nav1.8 conductance is sufficient to antagonize current clamp metrics associated with hyperexcitability. As a primary determinant of efficacy, we evaluated the ability of the Nav1.8 SLiP to reduce DRG neuron excitability by quantifying the total number of action potentials elicited following a suprathreshold current injection (Figure 4A, left). In basal conditions, there was no significant difference in the total number of action potentials between groups (Scram SLiP 2.5 ± 0.5 and Nav1.8 SLiP 1.4 ± 0.2). We then investigated whether the Nav1.8 SLiP impacted rheobase (Figure 4B) but there was no significant difference between groups either (Scram SLiP 180.0 ± 22.7 pA and Nav1.8 SLiP 189.2 ± 27.9 pA). A similar trend was observed for whole-cell capacitance (Figure 4C; Scram SLiP 18.66 ± 1.416 and Nav1.8 SLiP 19.6 ± 1.7 pF). However, there was a significant difference in resting membrane potential (Figure 4D) where the Nav1.8 SLiP group (−52.2 ± 0.8 mV) was slightly more depolarized compared to the Scram SLiP group (−54.6 ± 0.5 mV). We then quantified different aspects of the action potential (Figure 4E), including overshoot (Figure 4F; Scram SLiP 58.5 ± 1.2 mV and Nav1.8 SLiP 52.6 ± 1.5 mV), threshold (Figure 4G; Scram SLiP −22.7 ± 1.4 mV and Nav1.8 SLiP −20.5 ± 1.6 mV), and half-width (Figure 4H; Scram SLiP 2.0 ± 0.1 ms and Nav1.8 SLiP 2.1 ± 0.1 ms). These data suggest that while the Nav1.8 SLiP does displace Nav1.8 from Ank3 in basal conditions, as evident by a decrease in action potential overshoot and resting membrane potential, it is without substantial electrophysiological consequence.

**Figure 4:**
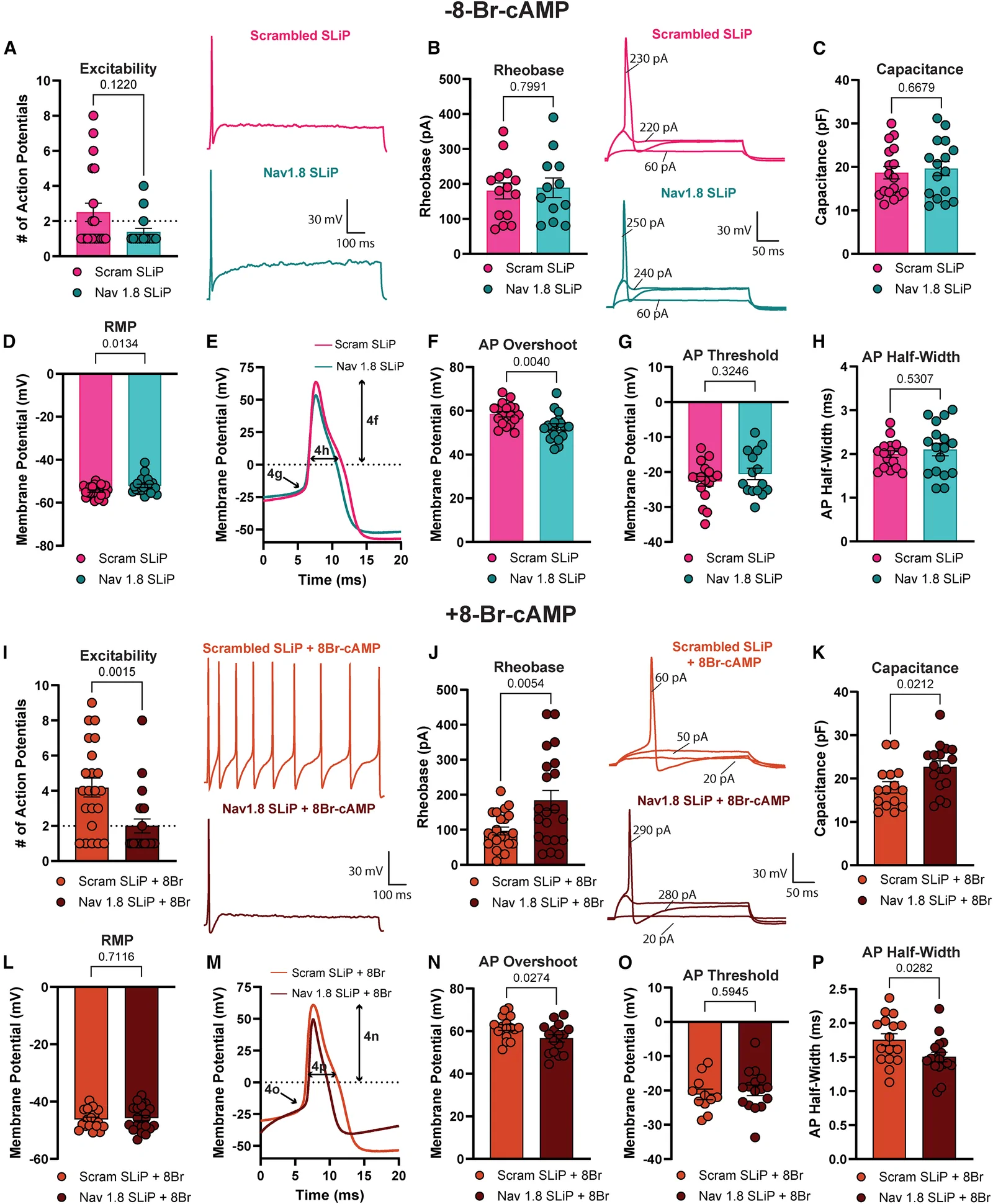
Nav1.8 SLiP decreases current clamp metrics associated with Nav1.8 in a PKA-dependent manner (A–H) Current clamp recordings of eDRG neurons in the basal/physiological state. (A) (Left) Total number of action potentials elicited following a suprathreshold stimulus (2× rheobase) for 1 s (Scram SLiP; *n* = 20, Nav1.8 SLiP; *n* = 16). (Right) Representative excitability traces. (B) (Left) Rheobase of recorded eDRG neurons from each condition (Scram SLiP; *n* = 14, Nav1.8 SLiP; *n* = 12). (Right) Representative rheobase traces. (C) Whole-cell capacitance of recorded eDRG neurons (Scram SLiP; *n* = 17, Nav1.8 SLiP; *n* = 16). (D) Resting membrane potential of recorded eDRG neurons (Scram SLiP; *n* = 23, Nav1.8 SLiP; *n* = 21). (E) Peak aligned representative action potential curves. (F) Action potential amplitude above 0 mV for each condition (Scram SLiP; *n* = 18, Nav1.8 SLiP; *n* = 18). (G) Threshold potential for action potential generation in recorded neurons (Scram SLiP; *n* = 17, Nav1.8 SLiP; *n* = 15). (H) Action potential half-width (Scram SLiP; *n* = 17, Nav1.8 SLiP; *n* = 17). (I–P) Current clamp recordings of eDRG neurons following incubation with 250 μM 8-Br-cAMP (pathological state). (I) (Left) Total number of action potentials elicited following a suprathreshold stimulus (2× rheobase) for 1 s (Scram SLiP; *n* = 22, Nav1.8 SLiP; *n* = 21). (Right) Representative excitability traces. (J) (Left) Rheobase of recorded neurons from each condition (Scram SLiP; *n* = 22, Nav1.8 SLiP; *n* = 21). (Right) Representative rheobase traces. (K) Whole-cell capacitance of recorded eDRG neurons (Scram SLiP; *n* = 15, Nav1.8 SLiP; *n* = 16). (L) Resting membrane potential of recorded eDRG neurons (Scram SLiP; *n* = 20, Nav1.8 SLiP; *n* = 22). (M) Peak aligned representative action potential curves. (N) Action potential amplitude above 0 mV for each condition (Scram SLiP; *n* = 16, Nav1.8 SLiP; *n* = 16). (O) Threshold potential for action potential generation in recorded neurons (Scram SLiP; *n* = 11, Nav1.8 SLiP; *n* = 16). (P) Action potential half-width (Scram SLiP; *n* = 16, Nav1.8 SLiP; *n* = 17). For all data presented, data were pooled from five independent experiments. For (A) and (I), statistical significance determined using the Mann-Whitney nonparametric test. For all other comparisons, significance determined using a two-tailed unpaired Student’s *t* test. All data presented as mean ± SEM.

However, when repeating the abovementioned experiments in the presence of 8-Br-cAMP, significant changes were observed. First, there was a significant reduction in the total number of action potentials elicited following a suprathreshold stimulus (Figure 4I, left; Scram SLiP 4.2 ± 0.5 and Nav1.8 SLiP 2.0 ± 0.4). There was also a significant increase in rheobase (Figure 4J; Scram SLiP 96.4 ± 11.1 pA and Nav1.8 SLiP 184.0 ± 28.3 pA) and whole-cell capacitance (Figure 4K; Scram SLiP 18.0 ± 1.3 pF and Nav1.8 SLiP 22.7 ± 1.4 pF) without affecting resting membrane potential (Figure 4L; Scram SLiP −46.2 ± 0.7 mV and Nav1.8 SLiP −45.7 ± 0.9 mV), suggesting that the excitability metrics dominated by other ion channels were left unchanged. Additionally, there were differences in various metrics associated with the action potential profile, namely overshoot (Figure 4N; Scram SLiP 61.7 ± 1.4 mV and Nav1.8 SLiP 56.7 ± 1.7 mV) and half-width (Figure 4P; Scram SLiP 1.8 ± 0.1 ms and Nav1.8 SLiP 1.5 ± 0.1 ms), while threshold (Figure 4O; Scram SLiP −21.2 ± 1.6 mV and Nav1.8 SLiP −20.0 ± 1.5 mV) remained similar between both groups. Altogether, these data show that pharmacological inhibition of Nav1.8/Ank3 binding reduces hyperexcitability metrics associated with Nav1.8 in sensory neurons in a PKA-dependent manner.

### Nav1.8 SLiP reduces calcium entry when applied to either the soma or axon

Next, we wanted to determine whether the Nav1.8 SLiP affects calcium entry, a functional metric of excitability/depolarization, when applied to somatic and axonal compartments.

When the Nav1.8 SLiP was applied to the entire neuron in the presence of 8-Br-cAMP (Figure 5A), there was a significant decrease in somatic calcium flux (Figure 5B), peak calcium flux (Figure 5C), and total calcium flux (Figure 5D). Then we investigated whether the Nav1.8 SLiP modulates calcium entry in a similar manner if applied directly to the axon. To do this, we utilized microfluidic chambers to separate the axonal and somatic compartments (Figure 5E). Axonal application of the Nav1.8 SLiP, in the presence of 8-Br-cAMP, resulted in a decrease in somatic calcium flux (Figure 5F), peak calcium flux (Figure 5G), and total calcium flux (Figure 5H). The data show that the Nav1.8 SLiP reduces calcium flux due to a reduction in Nav1.8 conductance when applied in either somatic or axonal compartments.

**Figure 5:**
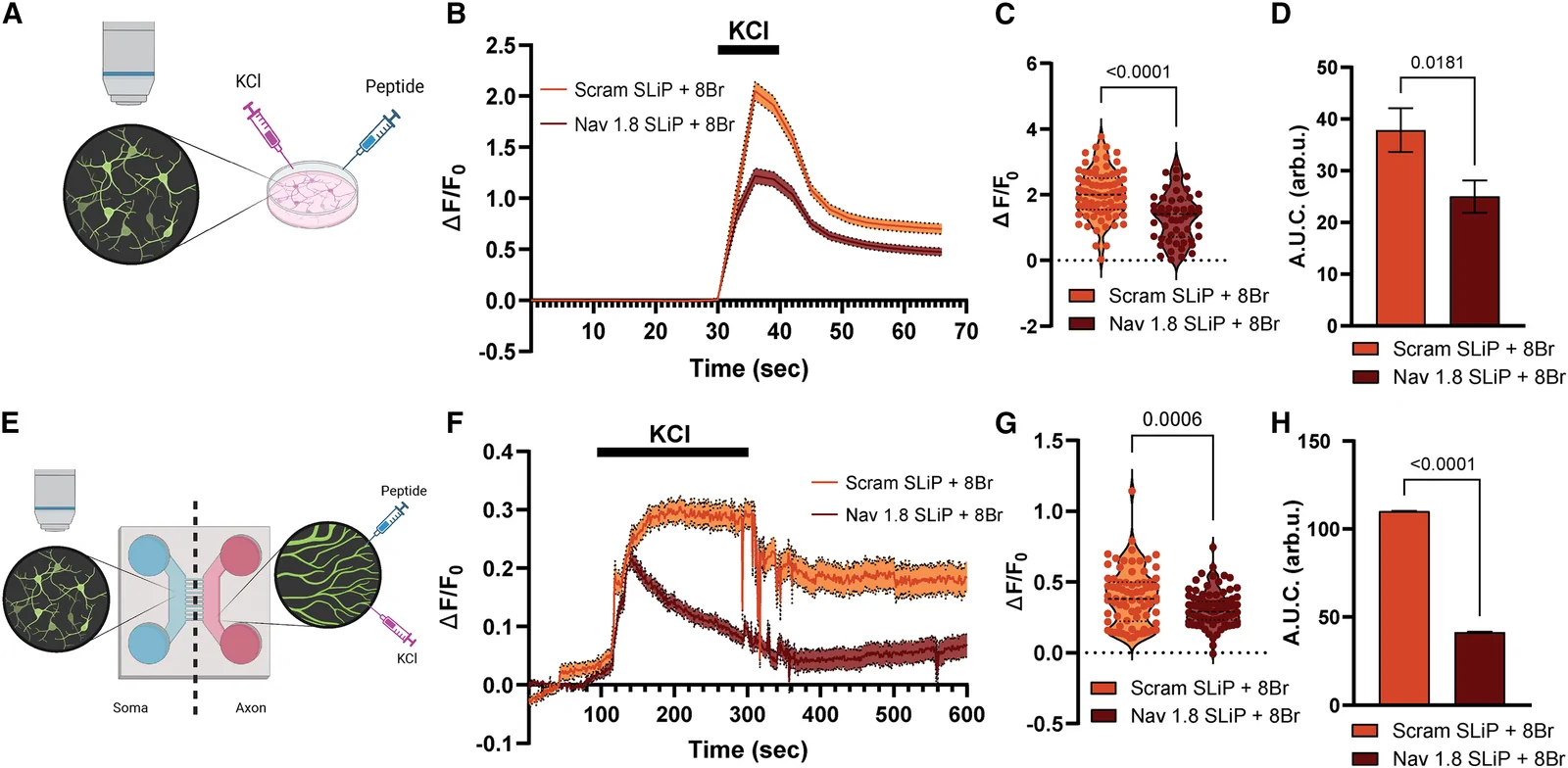
Nav1.8 SLiP decreases calcium flux when applied directly onto the soma or axons of sensory neurons in the presence of 8-Br-cAMP (A–D) Calcium imaging experiments where peptides were added to the entire cell (bath incubation) in the presence of 8-Br-cAMP. (A) Schematic of bath incubation calcium imaging paradigm. (B) Average change in fluorescence for cells sensitized with 8-Br-cAMP with either the Scram (*n* = 70) or Nav1.8 SLiP (*n* = 64) following depolarization with KCl. (C) Peak change in fluorescence of curves presented in (B). (D) Area under the curves presented in (B). (E–H) Calcium imaging experiments where peptides, 8-Br-cAMP, and KCl were applied axonally. (E) Schematic of microfluidic calcium imaging paradigm. (F) Average change in fluorescence for cells and peptides (Scram SLiP *n* = 76, Nav1.8 SLiP *n* = 95) following axonal depolarization with KCl. (G) Peak change in fluorescence of curves presented in (F). (H) Area under curves presented in (G). All data shown are pooled replicates from four independent experiments. For (B) and (F), statistical significance was determined using two-way ANOVA with multiple comparisons and Holm-Šídák post-hoc correction. For all other statistical comparisons, significance was determined using a two-tailed unpaired Student’s *t* test. All data presented as mean ± SEM. Illustrative graphics were created in BioRender. Powell, R. (2026) https://BioRender.com/7losi45.

Interestingly, when the Nav1.8 SLiP was added to the entire cell in the basal/physiological state, there was unexpectedly a significant increase in calcium flux compared to the Scram SLiP control (Figure S6A). However, the Nav1.8 SLiP had no effect on calcium entry into axons in the basal/physiological state (Figure S6D) nor did it affect peak calcium entry (Figure S6E) or total calcium entry (Figure S6F).

### Nav1.8 SLiP reduces pain-like behaviors in a model of neuropathic pain

To verify if the *in vitro* results translated into *in vivo* models of pain, we evaluated the efficacy of the Nav1.8 SLiP in a neuropathic pain model. We chose a chronic neuroma model of persistent pain with late-onset, stimulus-evoked and spontaneous-pain-like behaviors, which are stable for months.^67^

First, we characterized the proteomic landscape of the neuroma bulb and identified proteins that exhibited significant changes in expression when compared to uninjured sciatic nerves (Figure 6A). Ank2 and Ank3 were among the upregulated proteins, while Ank1 did not display increased expression. In addition, the alpha and beta catalytic subunits of PKA (*Prkaca* and *Prkacb*) were also significantly upregulated, while other cAMP-binding proteins (*Rapgef2* and *Rapgef4*) were not. We used immunofluorescence to confirm the presence of Nav1.8 and Ank3 in the neuroma bulb (Figure 6B).

**Figure 6:**
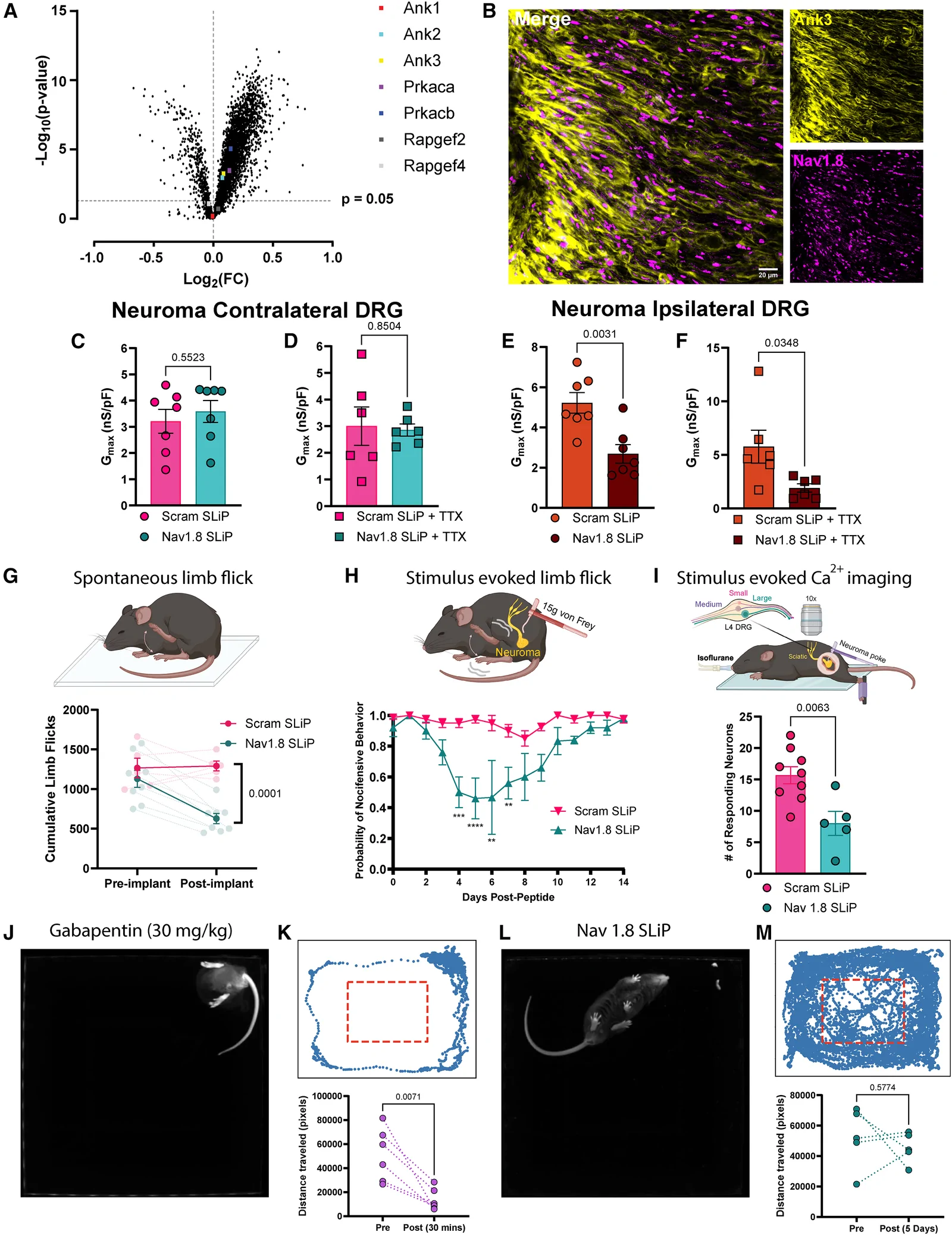
Reducing the Nav1.8-Ank3 reduces *in vitro* and *in vivo* metrics associated with a neuroma model of neuropathic pain while sparing mobility (A) Volcano plot highlighting proteins of interest with changes in expression in neuromas taken from high neuroma pain mice compared to contralateral uninjured nerves (pooled from nine males and nine females). (B) Representative immunofluorescent images showing presence of Nav1.8 and Ank3 in a neuroma bulb. Scale bar is 20 μm in length. (C–D) Maximum Nav channel conductance from aDRG neurons contralateral to a painful neuroma injury. (C) Maximum Nav channel conductance in aDRG neurons incubated with either Scram SLiP (*n* = 7) or Nav1.8 SLiP (*n* = 7). (D) Maximum conductance of TTX-R Nav channels in aDRG neurons incubated with either Scram SLiP (*n* = 6) or Nav1.8 SLiP (*n* = 6). (E–F) Maximum Nav channel conductance from aDRG neurons ipsilateral to a painful neuroma injury. (E) Maximum Nav channel conductance in aDRG neurons incubated with either Scram SLiP (*n* = 7) or Nav1.8 SLiP (*n* = 7). (F) Maximum conductance of TTX-R Nav channels in aDRG neurons incubated with either Scram SLiP (*n* = 6) or Nav1.8 SLiP (*n* = 6). (G–I) Effect of Nav1.8 SLiP on spontaneous and evoked pain-related behaviors and DRG activity in the neuroma model. (G) Number of spontaneous limb flicks before cuff implantation and 5 days after implantation (Scram SLiP; *n* = 5 [2 males and 3 females], Nav1.8 SLiP; *n* = 8 [5 males and 3 females]). (H) Probability of behavioral response following cutaneous mechanical stimulation of a neuroma before and after application of peptides by a cuff at day 0 (Scram SLiP; *n* = 4 [3 males and 1 female], Nav1.8 SLiP; *n* = 5 [2 males and 3 females]). (I) Number of nociceptors responding to mechanical neuroma stimulation (Scram SLiP; *n* = 9 mice [3 males and 6 females], Nav1.8 SLiP; *n* = 5 mice [2 males and 3 females]). (J–M) Comparison of animal mobility at peak analgesia between gabapentin and Nav1.8 SLiP in neuroma model of neuropathic pain. (J) Representative still image of a mouse 30 min after gabapentin administration. (K) (Top) Movement map depicting movement pattern of mouse from (J) during a 20-min recording session 30 min after gabapentin administration. (Bottom) Quantification of total distance traveled by mice before and after gabapentin administration (*n* = 6; [4 males and 2 females]). (L) Representative still image of a mouse 5 days after Nav1.8 SLiP administration. (M) (Top) Movement map depicting movement pattern of mouse from (L) during 20-min recording session 5 days after Nav1.8 SLiP administration. (Bottom) Quantification of total distance traveled by mice before and after Nav1.8 SLiP administration (*n* = 5; [2 males and 3 females]). For (C) and (D), statistical significance was determined using an unpaired Student’s *t* test. For (G), (K), and (M), statistical significance was determined using a paired *t* test. For (H), statistical significance was determined by two-way ANOVA with multiple comparisons and Holm-Šídák post-hoc correction. For (I), statistical significance was determined using an unpaired Student’s *t* test: ∗*p* < 0.05; ∗∗*p* < 0.01; ∗∗∗*p* < 0.005; ∗∗∗∗*p* < 0.0001. All data presented as mean ± SEM. Illustrative graphics created in BioRender. Powell, R. (2026) https://BioRender.com/nec73nn.

Next, we measured the efficacy of the Nav1.8 SLiP in neurons cultured from DRGs contralateral and ipsilateral to the neuroma. Unsurprisingly, the Nav1.8 SLiP had no effect on total Nav channel G_max_ (Figure 6C; Scram SLiP 3.2 ± 0.5 nS/pF and Nav1.8 SLiP 3.6 ± 0.4 nS/pF) in contralateral aDRG neurons. A similar trend was observed in the TTX condition, where G_max_ remained comparable between groups (Figure 6D; Scram SLiP 3.0 ± 0.7 nS/pF and Nav1.8 SLiP 2.9 ± 0.2 nS/pF). Conversely, the Nav1.8 SLiP precipitated effects in aDRG neurons isolated ipsilateral to the neuroma as evidenced by a significant reduction in G_max_ (Figure 6E; Scram SLiP 5.2 ± 0.5 nS/pF and Nav1.8 SLiP 2.7 ± 0.5 nS/pF). Isolating the TTX-R Nav channels continued the established trend with a reduction in G_max_ (Figure 6F; Scram SLiP 5.8 ± 1.5 nS/pF and Nav1.8 SLiP 1.9 ± 0.4 nS/pF). Altogether, these data suggest that the Nav1.8 SLiP reduces the Nav1.8 current in injured neurons in a manner similar to that observed in eDRG neurons.

With the confirmation that the Nav1.8 SLiP retains efficacy in cultured injured neurons, we next evaluated the efficacy of the Nav1.8 SLiP across pain-like behaviors and metrics associated with the neuroma model. First, we quantified the number of spontaneous hindlimb flicking bouts ipsilateral to the neuroma as a measure of spontaneous neuropathic pain.^67^ Animals administered the Nav1.8 SLiP locally via a perineural cuff implanted around the neuroma lesion showed a significant reduction in spontaneous limb-flicking bouts compared with pre-implantation levels (Figure 6G). The same approach also reduced responses to transcutaneous mechanical stimulation of the neuroma, with the reduction first emerging on day 4 post-implantation and remaining significant through day 7 (Figure 6H). The effects of the peptide were further characterized by *in vivo* calcium imaging of sensory neurons in the ipsilateral L4 DRG while directly stimulating the surgically exposed neuroma. In mice with the Nav1.8 SLiP-cuff, there was a significant decrease in the total number of small diameter neurons (soma diameter <25 μm) responding to mechanical neuroma stimulation at 5 days post-treatment (Figure 6I). In addition to efficacy, we also compared the Nav1.8 SLiP to gabapentin, a common pharmacological intervention for treating neuropathic pain in the clinic. Because gabapentin can cause sedation at analgesic doses,^68^^,^^69^^,^^70^ we sought to compare the locomotor effects of both approaches at their respective peak efficacy (Figures 6H and S7). As expected, gabapentin produced a significant reduction in locomotion compared with pre-injection levels (Figure 6K), while the Nav1.8 SLiP did not affect animal locomotor activity at its peak efficacy (Figure 6M). These findings highlight the feasibility of SLiP administration for dampening neuropathic pain without causing unwanted locomotor side effects.

The reduction in pain-like behaviors was not restricted to neuroma pain. We observed similar reductions in pain-like behaviors in a zymosan model of acute inflammatory pain and in the spared nerve injury (SNI) model of chronic neuropathic pain. In both these models, intraplantar injection of the Nav1.8 SLiP ipsilateral to the insult precipitated reductions in pain-like behaviors associated with their respective injury types. In the zymosan model, animals displayed reductions in thermal sensitivity (Figure S8A) and mechanical allodynia (Figure S8B). In the SNI model, mice displayed reductions in both mechanical sensitivity (Figure S8C) and cold allodynia (Figure S8D). To complete our understanding of the Nav1.8/Ank3 interaction, we finally tested the efficacy of the peptide in uninjured animals (Figure S8E). Injection of the Nav1.8 SLiP did not have any effect on mechanical withdrawal threshold 24 and 48 h post-injection. This suggests that the peptide only affects pathological pain.

These data demonstrate the efficacy of the Nav1.8 SLiP in two models of chronic neuropathic pain and in a model of acute inflammatory pain, highlighting the potential of the Nav1.8/Ank3 axis as a pharmacologically exploitable target for future analgesic drug development.

## Discussion

Pharmacologically accessing specific voltage-gated sodium channels for the treatment of chronic pain has proven to be a difficult task. In some cases, insufficient blockade of the target channel due to pharmacokinetic/pharmacodynamic barriers or severe adverse effect profiles have limited clinical implementation. However, preclinical models have consistently pinpointed Nav channel inhibition as a powerful modulator of pain. We adapted a sodium-channel-focused approach by targeting the key pain-triggering sodium channel Nav1.8, but instead of blocking it, we explored avenues to modulate its membrane expression. Using a Nav1.8 SLiP, we uncovered a protein-protein interaction between Nav1.8 and Ank3 that underlies injury-induced PKA-dependent hyperexcitability in nociceptors reinforcing the need to evaluate sensory neuron protein-protein interactions both in quiescent and hypersensitized states.

Our first major finding relates to the differential expression of the three ankyrin isoforms in the periphery. Ankyrins engage in protein-protein interactions that maintain electrogenesis in electrically excitable tissues through direct and indirect interactions with ion channels, extracellular matrix proteins, and intracellular cytoskeletal elements.^33^^,^^44^^,^^48^^,^^51^^,^^52^^,^^60^ In the CNS, Ank3 is highly concentrated at the axon initial segment (AIS) and in Nodes of Ranvier, where it organizes ion channels in the AIS that are critical for action potential generation.^49^^,^^50^^,^^71^^,^^72^ In nociceptors, action potentials can be generated at their AIS or in peripheral axon terminals requiring highly structured subcellular organization of Nav channels near the soma and in the periphery. Through immunofluorescent staining, we observed a high degree of Ank3 expression in Nav1.8^+^ axon terminals (Figure 1D), which suggests the subcellular organization observed in the AIS may be replicated in nociceptor axon terminals. In peripheral tissues, nociceptor terminals have a spike-initiation zone (SIZ), comprised of a Nav-independent and Nav-dependent zone, that is responsible for peripheral action potential generation.^73^ Interestingly, the SIZ is a dynamic substructure with expansion of the Nav-dependent zone following a noxious insult. Since ankyrins help stabilize ion channels in distinct subcellular domains,^45^^,^^52^ we propose that ankyrins may be a key contributor to SIZ lengthening in peripheral nociceptor fibers in pathological conditions as evidenced by our immunofluorescent staining.

Additionally, our data do not indicate that the ankyrin isoforms are interchangeable for Nav1.8 stabilization in PKA-stimulated conditions although they are co-expressed in sensory neurons, and we show that Nav1.8 is able to bind to all three (Figure S1D). This is highlighted by the reduction in Nav1.8 conductance not being adequately compensated by Ank1 or Ank2, even when knockdown of Ank3 resulted in significant increases in Ank1 and Ank2 mRNA (Figure 2D). The effects of Ank1 and Ank2 knockdown on total G_max_ may result from interactions with other Nav channels expressed in nociceptors,^74^^,^^75^ while Ank3 serves as the obligatory ankyrin responsible for binding to and stabilizing Nav1.8. However, the exact mechanism of how all three ankyrin isoforms work in concert to contribute to nociceptor excitability is complicated by numerous ankyrin splice isoforms and post-translation modifications that can modify protein localization and function (Figure S11). All three ankyrin isoforms display varying degrees of phosphorylation/dephosphorylation after injury; however, only Ank3 contains a putative PKA phosphorylation site. This invites further inquiry into the contributions of each Ank3 post-translational modification to Nav1.8 trafficking and stabilization.

Interestingly, Ho et al. have reported no change in thermal behavior in uninjured Advilin^Cre^-Ank3^fl/fl^ animals.^60^ There is also evidence that deletion of the ABD of Nav1.8 does not impair its localization to the surface of DRG soma or its trafficking along the axons under basal conditions.^76^ We found a similar phenomenon in our *in vitro* shRNA-mediated knockdown experiments, where knockdown of Ank3 had no effect on sodium channel conductance in basal conditions (Figures 2E and 2F). However, using a cell-permeable cAMP kinase activator, 8-Br-cAMP, to promote hyperexcitability in our eDRG culture system (Figure S3), we uncovered a functional role of ankyrins, specifically Ank3, in sensory neurons (Figures 2G and 2H). In the hyperexcitable state, knockdown of each ankyrin isoform reduced total sodium conductance (Figure 2G), but only Ank3 knockdown significantly reduced Nav1.8 conductance (Figure 2H). A reduction in G_max_ in the presence of elevated PKA activity suggests that ankyrins engage in Nav channel trafficking and/or stabilization in the membrane—a finding that is in agreement with previous observations in other cell types.^41^^,^^42^^,^^50^^,^^60^^,^^71^ However, this begs the question “why is this effect only observable in the presence of 8-Br-cAMP?” Among the Nav channels, Nav1.8 is sensitive to PKA phosphorylation at an ER-retention motif, “RRR,” that increases Nav1.8 trafficking to the membrane.^23^ Nav1.8 is also extremely sensitive to endocytosis^26^^,^^77^^,^^78^^,^^79^^,^^80^ through interactions with ubiquitin ligases and endocytic machinery. Previous studies have shown that endocytosis is necessary to drive nociceptor excitability through ion channel redistribution at the membrane,^57^ and by inhibiting endocytosis, nociceptors are unable to fully hypersensitize.^56^ We have expanded on this idea by highlighting Ank3’s ability to protect Nav1.8 from endocytosis during sustained pain signaling. Antagonism of the Nav1.8/Ank3 interaction either genetically (Figure 2) or pharmacologically (Figure 3) produced a clear reduction in Nav1.8 conductance, which affects nociceptor excitability. This is further strengthened by the lack of efficacy of the Nav1.8 SLiP when clathrin-mediated endocytosis was inhibited by PitStop2 (Figure S10). These data support the role of Ank3 as a Nav1.8-binding protein responsible for stabilizing its membrane expression in a dynamic subcellular environment.

Next, we aimed to evaluate the pharmacological validity of the Nav1.8/Ank3 interaction using a Nav1.8 SLiP to disrupt its interaction with Ank3 (Figure S4). In the presence of 8-Br-cAMP, we show that our Nav1.8 SLiP exerts a neurobiologically relevant effect in a manner like that observed in the Ank3 shRNA knockdown. Unexpectedly, the SLiP we used (Figure 3B) is less than half the length used in Montersino et al. but only affected Nav1.8 conductance (Figures 3F and 3H). This is interesting because the chosen sequence does not include CK2 phosphorylation sites (Figure S9)—sites that are necessary for increasing Nav channel affinity for ankyrins—^81^ revealing that this minimalistic sequence retains intrinsic specificity potentially brought about by the glutamic acid in the variable region bookended by the ABD. When aligning the ABD of all Nav channels, it is apparent that there are differences in the variable regions between many of the Nav channels; however, the consequence of these and how they affect each Nav channels relationship with the ankyrins has yet to be fully explored.

Since action potentials serve as the biochemical “currency” of the nervous system, and its frequency functions as a correlate for pain, the observed reductions in current clamp metrics (Figure 4) provided an initial indicator that inhibiting the Nav1.8/Ank3 interaction may reduce pain-like behaviors *in vivo.* We utilized the Nav1.8 SLiP in both acute and chronic inflammatory and neuropathic pain models, and it precipitated alterations in pain-like behaviors in all instances, except in acute nociceptive pain (Figures 6 andS8). By applying the Nav1.8 SLiP locally at the site of insult, we observed a profound decrease in pain-like behavior, suggesting that the Nav1.8/Ank3 interaction is not just limited to nerve injury, but a process commonly engaged during different forms of pathological pain. This highlights that nociceptors leverage highly compartmentalized interactions to facilitate pathological nociceptive signaling. However, this raises questions about the mechanism of the Nav1.8 SLiP and, by extension, the importance of Nav1.8/Ank3 interactions. The Bhattacharjee lab has described how nociceptors utilize endocytosis to remove large conductance sodium-activated potassium channels to facilitate high-frequency firing in models of pain.^24^^,^^26^^,^^56^^,^^57^ Interestingly ankyrins antagonize the endocytosis of their bound cargo by imposing membrane rigidity in their immediate area.^45^^,^^57^^,^^82^^,^^83^ Therefore, it is not surprising that the electrophysiological effects of the Nav1.8 SLiP could be reversed by inhibiting endocytosis (Figure S10). This suggests that the Nav1.8 SLiP displaces Nav1.8 from Ank3, making it vulnerable to endocytosis. This expectation was met when we applied the SLiP to peripheral tissues in two models of neuropathic pain (Figures 6 and S8), where we observed robust reductions in both stimulus-evoked and spontaneous pain metrics, as predicted by both electrophysiology and calcium flux imaging. These data support the observations made in Vasylyev et al.^29^ where they showed a partial reduction of Nav1.8 conductance (25%–50%) can reverse metrics associated with hyperexcitability (i.e., rheobase and spontaneous firing) in DRG neurons expressing a Nav1.7 gain-of-function mutation associated with pain in humans. Our data support their findings and highlight the role Nav1.8 plays in nociception in rodents. Nevertheless, by disrupting the Nav1.8/Ank3 interaction with our Nav1.8 SLiP, we clearly convert hyperexcitable cells back to their quiescent state, emphasizing the importance of this process for pathological pain signaling in nociceptors.

Lastly, we would like to highlight that these findings may have translational potential. In painful human neuromas, an increased presence of kinases,^84^ Nav1.8,^22^ and Ank3^85^ have been documented. More evidence that this interaction is of importance for maintaining pathological pain signaling across species is now required.

Nav1.8 and its ability to bind ankyrins has led to the discovery of a state-dependent interaction between Nav1.8 and Ank3. Our work provides identification of the three ankyrin isoforms in various locations in sensory neurons, electrophysiological characterization of the consequence of ankyrin isoform knockdown, SLiP-based pharmacology, and confirmation of the *in vitro* findings in *in vivo* pain models. These experiments reveal that peripheral sensory neurons recontextualize protein-protein interactions during persistent pain signaling, offering a strategy to selectively reduce pathological pain without impairing the protective role of acute nociceptive pain.

### Limitations of the study

Here, we describe the consequence of disrupting Nav1.8/ankyrin interactions in sensory neurons *in vitro* and *in vivo*. There exist, however, some limitations in the presented experiments—the first being the electrophysiological recording experiments. All the recordings were conducted in neurons, which were kept in culture for extended periods of time (eDRGs 9–10 DIV and aDRGs 2 DIV) at which point they begin to sprout neuronal processes across the dish. These projections from the soma make voltage clamping extremely difficult if not accounted for with (1) reduced extracellular NaCl and (2) a pre-pulse protocol to inactivate axonal sodium channels to isolate somatic Nav currents. This space-clamp artifact reduces data resolution, resulting in inappropriate I/V relationships and residual voltage errors. In aDRGs, this artifact is further exacerbated by their larger average size and larger currents. As a result, we chose to only report maximum conductance and not I/V curves to minimize space clamp issues from skewing data interpretation. Work by Milescu, Bean, and Smith^86^ showed that poor space clamp strongly impacts the sodium channel I/V relationship at hyperpolarized potentials while not strongly impacting peak current. Additionally, some of the behavioral experiments have a low number of replicates. We did not observe any significant differences in responses between male and female animals, so they were pooled together. However, sex does serve as a biological variable, and a properly powered experiment may uncover sex-dependent responses to the Nav1.8 SLiP that were not observed here. Additionally, there are limitations to the clinical relevance of these findings in humans. Human Nav1.8 and Ank3 share above 80% amino acid identity with their respective mouse analogs. The core ABD in hNav1.8 is conserved across rodents to humans, so the Nav1.8/Ank interaction may be similar to that observed in rodent nociceptors; however, this requires experimental validation.

## Resource availability

### Lead contact

Requests for further information and resources should be directed to, and will be fulfilled by, the lead contact, Clifford J. Woolf (clifford.woolf@childrens.harvard.edu).

### Materials availability

This study did not generate new unique reagents.

### Data and code availability

• Proteomic data have been deposited to the ProteomeXchange Consortium via the PRIDE partner repository^87^ as dataset identifier PXD082960 and are publicly available. Raw data supporting the findings of this study will be shared by the lead contact upon request.
• This paper does not report original code.
• Any additional information required to reanalyze the data reported in this work paper is available from the lead contact upon request.

## Acknowledgments

We thank Drs. Bruce P. Bean, Arin Bhattacharjee, and Matthew Rasband for their constructive feedback and expertise in guiding this research. We also thank the BCH Cellular Imaging Core (RRID: SCR_026485), supported by funding from the National Institutes of Health (10.13039/100000002NIH P50 HD105351). Schematics and illustrations were generated using BioRender.com. Structural predictions were generated using Neurosnap.ai and modeled using Pymol. This work was supported by funding from the 10.13039/100000002National Institutes of Health (NIH R35 NS105076) and the Dr. Miriam and Sheldon G Adelson Medical Research Foundation.

## Author contributions

R.P. and C.J.W. conceived the study. R.P. performed all electrophysiological, immunofluorescent, and biochemical experiments and analyzed the data. X.Z. conducted neuroma surgeries and conceived all experiments that derived from the neuroma model. B.L.T. conducted all SNI surgeries. J.A.O.-P. conducted proteomic experiments and analysis. V.P. conducted qPCR experiments and performed data analysis. H.W.K. prepared peripheral neuronal tissue and expertise for tissue staining. X.Z. and S.S.J. collected tissue for proteomic analysis. G.K. conducted microfluidic experiments and subsequent data collection and analysis. E.S. scored spontaneous behavior for neuroma experiments. K.H. stained neuroma tissue. M.A. conducted mechanical sensitivity testing and SNI experiments. E.C. conducted thermal sensitivity testing in the zymosan model. S.D.D.-H. and S.G.W. provided halo-tagged constructs and provided expertise and oversight for live cell imaging. A.L.B. provided oversight and expertise for proteomic analysis. C.J.W. oversaw the project. R.P. and C.J.W. wrote the manuscript with input from all authors.

## Declaration of interests

R.P. is the chief scientific officer and co-founder of Mimetic Medicines. C.J.W. is a founder of Nocion Therapeutics, Quralis, and BlackBox Bio. He is also a member of the scientific advisory boards of Lundbeck Pharma, Axonis, Tafalgie Therapeutics, Niroda, Mimetic Medicines, and Tenvie. R.P., C.J.W., and X.Z. declare that a patent application related to the work described in this manuscript has been filed through Boston Children’s Hospital. S.G.W. has served during the past 48 months as an advisor to Mimetic Medicines, SiteOne Therapeutics, Navega Therapeutics, Channel-Chromocell, OliPass Pharma, Latigo Therapeutics, Sangamo, Third Rock Ventures, Foresite Labs, Exicure, Arrowhead Pharmaceuticals, GenEP Therapeutics, Enveda Biosciences, Spark Therapeutics, Jazz Therapeutics, Alnylam, Population Health Partners, Medtronics, and Vertex Pharmaceuticals. S.D.D.-H. has served in the past 2 years as an advisor to SiteOne Therapeutics, GenEp Therapeutics, and Population Health Partners.

## STAR★Methods

### Key resources table

**Table:** 

REAGENT or RESOURCE	SOURCE	IDENTIFIER
**Antibodies**		

Goat anti-Ankyrin1, cat.no. MBS421627	MyBioSource	RRID: AB_2242823
Rabbit anti-Ankyrin1, cat. no. 23938-1-AP	Proteintech	RRID: AB_3085717
Mouse anti-Ankyrin2, cat.no. S105-13	Antibodies com	RRID: AB_2877371
Rabbit anti-Ank2, cat.no. BS-6967R	Bioss	RRID: AB_3753517
Mouse anti-Ankyrin3, cat.no. sc-12719	Santa Cruz Biotechnology	RRID: AB_626674
Rabbit anti-Ankyrin3, cat.no. PA5-143596	Thermo Fisher Scientific	RRID: AB_2942825
Mouse anti-Nav1.8, cat.no. 75-166	Antibodies Incorporated	RRID: AB_2183861
Rabbit anti-Nav1.8, cat.no. ASC-016	Alomone Labs	RRID: AB_2040188
Donkey anti-Goat 488, cat.no. 705-545-003	Jackson ImmunoResearch	RRID: AB_2340428
Donkey anti-Mouse 568, cat.no. A10042	Thermo Fisher Scientific	RRID: AB_11180865
Donkey anti-Rabbit Cy5, cat.no. 711-175-152	Jackson ImmunoResearch	RRID: AB_2340607
Donkey anti-Rabbit IRDye-680RD, cat.no. 926-68073	LICORBio	RRID: AB_10954442
Goat anti-Mouse IRDye-800CW, cat.no. 827-08364	LICORBio	RRID: AB_10793856

**Bacterial and virus strains**		

pAAV-PHP.s	VectorBuilder	N/A

**Biological samples**		

Embryonic mouse DRG neurons	This paper	N/A
Adult Mouse DRG neurons	This paper	N/A

**Chemicals, peptides, and recombinant proteins**		

Nav1.8 SLiP (Myr-GGVPIAEGESDLDE-COOH)	This paper	N/A
Scrambled SLiP (Myr-ELDEPGGVISAEDG-COOH)	This paper	N/A
L-15 media	Cytiva	Cat#: SH30525.01
DMEM/F12	Thermo Fisher Scientific	Cat#: 11320033
Neurobasal media	Thermo Fisher Scientific	Cat#: 21103049
HBSS [no Ca^2+^, no Mg^2+^, no phenol red]	Thermo Fisher Scientific	Cat#: 14175103
NP-40 Lysis buffer	Thermo Fisher Scientific	Cat #:J60766.AP
Fetal bovine serum	Thermo Fisher Scientific	Cat#: 16000044
Bovine serum albumin	Sigma-Aldrich	Cat#: A9205
Normal Donkey Serum	Jackson ImmunoResearch	Cat#: 017-000-121
Penicillin-streptomycin	Thermo Fisher Scientific	Cat#: 15070063
NGF 2.5S	Sigma-Aldrich	Cat#: N6009
GDNF	Thermo Fisher Scientific	Cat#: PHC7045
Ara-C (Cytarabine)	Cayman Chemical Company	Cat#: 16069
Poly-D-lysine hydrobromide	Sigma-Aldrich	Cat#: P7280
Poly-L-lysine hydrobromide	Sigma-Aldrich	Cat#: P2636
Laminin	Invitrogen	Cat#: 23017-015
Lipofectamine 3000	Thermo Fisher Scientific	Cat#: L3000001
HaloTag Alexa Fluor 660 ligand	Promega	Cat#: G8472
8-Br-cAMP sodium salt	TargetMol	Cat#: T6747
Fast SYBR Green Master Mix	Applied Biosystems	Cat#: 4385612
Fura2-AM	Thermo Fisher Scientific	Cat#: F1221
Fluo4-AM	Thermo Fisher Scientific	Cat#: F14217
Probenecid	Sigma-Aldrich	Cat#: P8761
Tetrodotoxin citrate	Tocris	Cat#: 1069
Zymosan	Sigma-Aldrich	Cat#: Z4250
cOmplete Protease inhibitor cocktail	Sigma-Aldrich	Cat#: 11697498001
Zwitterionic 3–16 detergent	Sigma-Aldrich	Cat#: 693023
Lysyl endopeptidase (LysC)	FUJIFILM	Cat#: 125-05061
TPCK-Trypsin	Thermo Fisher Scientific	Cat#: 20233

**Critical commercial assays**		

Pierce™ BCA Protein Assay Kit	Thermo Fisher Scientific	Cat#: 23227
SuperScript VILO cDNA Synthesis Kit	Thermo Fisher Scientific	Cat#: 11754050
Protein A/G magnetic beads	Selleck Chemical	Cat#: B23202
TMTProTM-18 label plex kit	Thermo Fisher Scientific	Cat#: A52045
5 μm Titanium dioxide (TiO2) beads	GL Sciences	Cat#: 5020-75000

**Deposited data**		

Proteomic and phosphoproteomic data	This paper	ProteomeXchange consortium via PRIDE (ID: PXD082960)

**Experimental models: Organisms/strains**		

Mouse: C57BL/6J [Strain #: 000664]	The Jackson Laboratory	RRID: IMSR_JAX:000664
Mouse: C57BL/6NCrl [Strain #: 027]	Charles River Laboratory	RRID: IMSR_CRL:027
Mouse: Scn10a-Cre [Strain #: 036564]	The Jackson Laboratory	RRID: IMSR_JAX:036564
Mouse: Ai14(RCL-tdT)-D [Strain #: 007914]	The Jackson Laboratory	RRID: IMSR_JAX:007914
Mouse: Thy-1 YFP-16 [Strain #: 003709]	The Jackson Laboratory	RRID: IMSR_JAX:003709
Mouse: Ai95(RCL-GCaMP6f)-D (C57BL/6J) [Strain #:028865]	The Jackson Laboratory	RRID: IMSR_JAX:028865
Mouse: Vglut2-ires-cre knock-in (C57BL/6J) [Strain #:028863]	The Jackson Laboratory	RRID: IMSR_JAX:028863

**Oligonucleotides**		

*Ank1* primers (Fwd 5′-TGCATGACGGATGACAAAGTGG-3′, Rev 5′-GGGATGGCCAGACGATTCTC-3′)	Stankewich et al.^56^	N/A
*Ank2* primers (Fwd 5′-GCCTCACCAAGATCAACAGG-3′, Rev 5′-GGCTGTCCCCTTCAGTTTTG-3′)	Stankewich et al.^56^	N/A
*Ank3* primers (Fwd 5′-CTGGTAAAGAGACATAAACTGGC-3′, Rev 5′-CCATTGAGAAGCTCCGCGAG-3′)	Stankewich et al.^56^	N/A
*Hprt1* primers (Fwd 5′-CAGTCCCAGCGTCGTGATTA-3′, Rev 5′-TGGCCTCCCATCTCCTTCAT-3′)	Jain et al.^57^	N/A

**Recombinant DNA**		

Nav1.7-halo-tag	Higerd-Rusli et al.^55^	N/A
Nav1.8-halo-tag	Higerd-Rusli et al.^55^	N/A
*Ank1* shRNA plasmid (shRNA sequence: 5′- CTTCCTATTGTGAAGAACTTA-3′)	VectorBuilder	N/A
*Ank2* shRNA plasmid (shRNA sequence: 5′- AGACATATCTGTCGGTTATTC-3′)	VectorBuilder	N/A
*Ank3* shRNA plasmid (shRNA sequence: 5′-CGGATAAGCTAGTCGATTATT-3′)	VectorBuilder	N/A
Control EGFP plasmid	VectorBuilder	N/A

**Software and algorithms**		

GraphPad Prism	GraphPad	RRID: SCR_002798
OriginPro 8.5	OriginLab	RRID: SCR_014212
NIS-Elements	Nikon	RRID: SCR_014329
Neurosnap	Neurosnap.ai	https://neurosnap.ai/
Fiji	Schindelin et al.^54^	RRID: SCR_002285
MultiClamp 700B Microelectrode command software	Molecular Devices	RRID: SCR_018455
Clampex 10.7	Molecular Devices	RRID: SCR_011323
PAVA software	Guan et al.^63^	N/A
Protein Prospector	Clauser et al.^64^	RRID: SCR_014558
Pylon Viewer	Basler	N/A
DeepLabCut	DeepLabCut	RRID: SCR_021391
Leica Application Suite X	Leica Microsystems	RRID: SCR_013673
PymoL	Schrödinger	RRID: SCR_000305

### Experimental model details

#### Animals

Wild-type mice used in the behavioral experiments and for tissue collection were purchased from The Jackson Laboratory (Bar Harbor, Maine; C57BL/6J Strain #: 000664, RRID: IMSR_JAX:000664). Pregnant C57BL/6NCrl dams at gestational day 11.5 were purchased from Charles River Laboratories (Wilmington, Massachusetts; Strain #: 027, RRID: IMSR_CRL:027). Nav1.8-Cre mice (The Jackson Laboratory; *Scn10a*-Cre, Strain#: 036564, RRID: IMSR_JAX:036564) were crossed with Ai14(RCL-tdT)-D reporter mice (The Jackson Laboratory; Strain#: 007914, RRID: IMSR_JAX:007914) to label peripheral nociceptive sensory neurons. The resulting Nav1.8-Cre;Ai14 offspring were further bred with *Thy1*-YFP-16 mice (The Jackson Laboratory; Strain #: 003709, RRID: IMSR_JAX:003709) to generate triple-positive mice, in which nociceptors were tdTomato+ and non-nociceptive sensory neurons were YFP+. There were no observable statistical differences between male and female mice, so male and female mice were pooled in all experiments and tissue collection. All mice were housed in the laboratory animal facility at Boston Children’s Hospital (BCH). All experiments were conducted with mice between 5–7 weeks. All mice were co-housed with age-matched littermates with a maximum of 5 mice per cage. Mice purchased for behavioral experiments were allowed 7 days to acclimate to the animal facility before experimentation. All the mice were housed in a temperature-controlled room with a 12-h light/dark cycle and had access to food and water *ad libitum*. All animal experimentation was conducted in accordance with the guidelines set by the Guide for the Care and Use of Laboratory Animals provided by the National Institutes of Health. All animal protocols were reviewed and approved by the Institutional Animal Care and Use Committee at BCH. Details for experiments using animals are outlined in the method details section.

#### Primary mouse dorsal root ganglion (DRG) neuron cultures

Sensory DRG neurons were harvested from both male and female animals for electrophysiology and imaging experiments. Neurons were randomly sampled across a dish during each experiment to minimize potential plating artifacts. Embryonic DRG experiments were conducted 9–10 days after plating, while adult DRG experiments were conducted 2 days after plating. Specific details for culturing conditions can be found in the method details section.

### Method details

#### Embryonic mouse DRG cultures

To collect embryonic DRG tissues (eDRG), a pregnant dam was sacrificed at gestational day 13.5 using CO_2_. Then a medial incision was made and the uterus housing the embryos was removed and placed into a dissection dish containing ice-cold PBS. In a separate dissection dish, the embryos were dissected one at a time in PBS. The collected DRG were then placed in a 15 mL conical vial containing PBS and kept on ice. At the end of the dissection, the harvested eDRGs were briefly centrifuged at 1000 RCF for 3 min. PBS was carefully removed and replaced with 4 mL 0.25% Trypsin. The DRGs were then gently rocked on a horizontal shaker in a cell incubator at 37°C with 5% CO_2_ for 45 min. Following digestion, equal amounts of warmed L-15 media (Cytiva, cat. no. SH30525.01) were added to the eDRG suspension and triturated with a fire-polished curved pipette until no visible clumps remained. Immediately following trituration, the eDRGs were centrifuged for 5 min at 2000 RCF to pellet the cells. After centrifugation, the media was carefully removed, and 5 mL of fresh L-15 media was added to the pellet. The pellet was triturated until the solution turned cloudy and immediately centrifuged at 1500 RCF for 5 min. The previous step was repeated once more. After centrifugation and media removal, 1 mL of warm eDRG growth media 89% DMEM/F12 (Thermo Fisher Scientific, cat. no. 11320033), 10% fetal bovine serum (FBS; Thermo Fisher Scientific, cat. no. 16000044), 1% penicillin-streptomycin (P/S; Thermo Fisher Scientific, cat. no. 15070063), 1μg/25mL nerve growth factor (NGF; Sigma-Aldrich, cat. no. N6009) was added. The pellet was triturated until the solution turned cloudy. Cell density was determined using an automatic cell counter and plated on 35 mm cell culture dishes (Corning, cat. no. 353001) pre-coated with poly-D-lysine (Sigma-Aldrich, cat. no. P7280) and laminin (Invitrogen, cat. no. 23017-015). 24 h after plating, a half media change was carried out. 48 h after plating, another half media change was conducted, using eDRG growth media supplemented with 3 μM Ara-C (Cayman Chemical Company, cat. no. 16069). Each subsequent half media change was conducted every 48 h with the eDRG growth media + Ara-C. Experiments were conducted 9–10 days after initial plating.

#### Adult mouse DRG cultures

DRGs from adult male and female C57BL/6J mice (aDRG) were aseptically dissected and isolated. The DRGs were then pooled and dissociated enzymatically with collagenase and trypsin and suspended in warmed L-15 media. The DRGs were triturated using a fire-polished curved pipette until the solution turned cloudy. The DRG suspension was briefly centrifuged for 5 min at 1000 rpm to collect the cells. The cell pellet was resuspended in 2 mL of warmed L-15 media. This cell suspension was carefully filtered through a 15% bovine serum albumin (BSA; Sigma-Aldrich, cat. no. A9205) gradient and spun in a tabletop centrifuge for 15 min at 1200 rpm. The resulting cell pellet was resuspended in 1 mL of warmed aDRG media containing 89% DMEM/F12, 10% FBS, 1% P/S and 5 ng/mL GDNF (Thermo Fisher Scientific, cat. no. PHC7045). Cell density was determined using an automatic cell counter, and the cells were plated at the desired density onto dishes pre-coated with PDL and laminin. All experiments with aDRGs were conducted 48 h post plating.

#### Preparation of short lipidated peptides (SLiPs)

SLiPs were synthesized by GenScript and shipped as lyophilized powder. SLiPs were dissolved in 20 μL DMSO to create a stock solution. Appropriate volumes of the DMSO stock solution were dissolved in sterile saline to generate 1 mM small volume aliquots for future testing. When needed for experiments, these small aliquots were brought to volume using either warm media or sterile saline for *in vitro* or *in vivo* experiments, respectively. For *in vitro* experiments, the final concentration used was 30 μM. For *in vivo* experiments, the final concentration used was 300 μM. Final DMSO concentration was <0.05%. All aliquots were stored at −80°C until used. Aliquots were discarded immediately after use to prevent freeze–thaw cycles.

#### Immunofluorescent staining

eDRG neurons were cultured and maintained on PDL/laminin coated round glass coverslips (MatTek Life Sciences, cat. no. PCS-1.5-15) in a 12-well cell culture plate. eDRG neurons were fixed using 4% paraformaldehyde for 10 min at room temperature. The cells were then washed 3× with PBS and subsequently permeabilized using 0.025% Triton X-100 in PBS for 5 min at room temperature. Then, the cells were blocked using a blocking solution containing 10% normal donkey serum (NDS) (Jackson ImmunoResearch, cat. no. 017-000-121) and 3% BSA in PBS for 1 h at room temperature. Primary antibodies Goat anti-Ank1 (MyBioSource, cat. no. MBS421627, RRID: AB_2242823; 1:500), Mouse anti-Ank2 (Antibodies.com, cat. no. S105-13, RRID: AB_2877371; 1:500), Rabbit anti-Ank3 (Thermo Fisher Scientific, cat. no. PA5-143596 RRID: AB_2942825; 1:500) or Mouse anti-Ank3 (Santa Cruz Biotechnology, cat. no. sc-12719, RRID: AB_626674; 1:500), Rabbit anti-Nav1.8 (Alomone Labs, cat. no. ASC-016, RRID: AB_2040188; 1:500), or Mouse anti-Nav1.8 (Antibodies Incorporated, cat. no. 75–166, RRID: AB_2183861; 1:500) were added to the blocking solution and incubated with the cells for 24 h at 4°C. After primary incubation, the cells were gently washed with PBS three times for 5 min each wash and incubated with secondary antibodies Donkey anti-Goat 488 (Jackson ImmunoResearch, cat. no. 705-545-003, RRID: AB_2340428; 1:1000), Donkey anti-mouse 568 (Thermo Fisher Scientific, cat. no. A10042, RRID: AB_11180865; 1:1000), or Donkey anti-Rabbit Cy5 (Jackson ImmunoResearch, cat. no. 711-175-152, RRID: AB_2340607; 1:1000) for 24 h at 4°C. Then, the cells were gently washed with PBS three times for 5 min each wash. Finally, the cells were mounted onto Superfrost Plus Microscope slides (Fisher Scientific, cat. no. 22-037-246) using Prolong Glass Antifade Mountant (Thermo Fisher Scientific, cat. no. P36980). Mounted samples were stored at 4°C.

For tissue collection, mice were sacrificed using CO_2_ and dissected. Tissues of interest were placed in 4% paraformaldehyde and incubated overnight at room temperature. Then they were cryoprotected by incubating them in 30% sucrose at room temperature until the samples sank to the bottom of the container. After cryoprotection, samples were placed in Tissue-Tek O.C.T. media (Sakura Finentek, cat. no. 4583) and frozen using dry ice. DRGs and sciatic nerves were cryosectioned at 15 microns and placed onto charged Superfrost Plus Microscope slides. Samples were allowed to dry for 2 h at room temperature before staining. Samples were blocked in 10% NDS, 3% BSA, and 0.4% Triton X-100 in PBS for 24 h at 4°C. Samples were incubated in primary and secondary antibodies as explained above. Finally, coverslips were mounted onto the sections using Prolong Glass Antifade Mountant. The sections were stored at 4°C until imaging.

All fluorescent images were imaged using a Leica TCS Laser Scanning Confocal Microscope (Leica Microsystems), preprocessed in Leica Application Suite X (LASX; Leica Microsystems, RRID: SCR_013673), and post-processed in Fiji.^88^

#### Live cell imaging

eDRG neurons were cultured as outlined above in glass bottom 35 mm dishes. On day 5, eDRG neurons were transfected with plasmids carrying either halo-tagged Nav1.7 or halo-tagged Nav1.8 constructs.^76^ Transfection was carried out using Lipofectamine 3000 (Thermo Fisher Scientific, cat. no. L3000001). The transfection was conducted following manufacturer’s instructions and 5 μg of DNA was used per dish.

After 3 days, the cells were incubated with the cell-impermeable HaloTag Alexa Fluor 660 ligand (Promega, cat. no. G8472) as per the manufacturer’s instruction with no modifications. After incubation with the halo-tag ligand, the cells were washed 2× with PBS then bathed in culture media without pH indicator. During imaging, cells were imaged in a CO2 incubation chamber affixed to the microscope stage. Images were taken every 10 s for 30 min. A single image was taken at time 0 to establish a baseline, then, immediately, 250 μM 8-Br-cAMP (TargetMol, cat. no. T6747) was added to the media. Imaging continued until the 30-min mark, at which point the experiment was completed. Images were taken with a Leica TCS Laser Scanning Confocal Microscope, preprocessed in LASX and post-processed in Fiji.

#### AAV shRNA mediated knockdown

Custom shRNA vectors were designed, manufactured, and packaged into an adeno-associated virus serotype PHP.S (pAAV-PHP.S) by VectorBuilder. Target sequences were designed to knockdown canonical and splice variants of each ankyrin isoform: *Ank1* 5′- CTTCCTATTGTGAAGAACTTA-3′, *Ank2* 5′- AGACATATCTGTCGGTTATTC-3′, and *Ank3* 5′-CGGATAAGCTAGTCGATTATT-3’. The control virus only contained EGFP. All vectors had EGFP as a marker for identification in electrophysiology experiments. For all experiments, pAAV-PHP.S particles were added at a final concentration of 3∗10^9^ gc/mL. Experiments were conducted 4–5 days after initial pAAV-PHP.S exposure.

#### cDNA synthesis and qPCR

cDNA synthesis from the obtained RNA samples was performed following the manufacturer’s instructions using the SuperScript VILO cDNA Synthesis kit (Thermo Fisher Scientific, cat. no. 11754050). Relative gene expression was determined by RT-qPCR using previously published gene-specific primer pairs against each ankyrin - *Ank1* (Fwd 5′-TGCATGACGGATGACAAAGTGG-3′, Rev 5′-GGGATGGCCAGACGATTCTC-3′), *Ank2* (Fwd 5′-GCCTCACCAAGATCAACAGG-3′, Rev 5′-GGCTGTCCCCTTCAGTTTTG-3′), *Ank3* (Fwd 5′-CTGGTAAAGAGACATAAACTGGC-3′, Rev 5′-CCATTGAGAAGCTCCGCGAG-3′).^89^ Expression levels were normalized to *Hprt1* (Fwd 5′-CAGTCCCAGCGTCGTGATTA-3′, Rev 5′-TGGCCTCCCATCTCCTTCAT-3′)^90^ using the 2-ΔCt method. Primers and individual samples were incubated with Fast SYBR Green Master Mix (Applied Biosystems, cat. no. 4385612). Fluorescence was measured on a 7500 Fast Real-time PCR system (Applied Biosystems).

#### Electrophysiology

Glass electrodes were generated using a horizontal pipette puller (Sutter Instruments, cat. no. P-97). For current clamp experiments, pipettes were fire-polished for resistances of 7–9 MΩ and for voltage clamp experiments, pipettes were fire-polished for resistances of 2–5 MΩ. All recordings were conducted such that each experimental group was recorded on the same day immediately following its appropriate control condition to minimize variations in recording conditions. Cells were selected based on 2 criteria: 1) smoothness of the membrane and 2) size (<30 μm). Giga ohm seals were first established and maintained for at least 1 min before slight negative pressure was used to gain electrical access to the cell. Seal integrity was tested 1 min after establishing a whole-cell patch by giving a 5 mV depolarizing stimulus. Sodium current recordings were collected in the whole-cell patch-clamp configuration. Series resistance compensation was maintained between 60–80% and series resistance was determined using the Multiclamp-700B software (Molecular Devices, RRID: SCR_018455). Cells that did not maintain a proper seal during recording, as evidenced by a >25% change in response to a 5 mV depolarizing pulse at the end of recording, were discarded from data analysis. For all sodium current recordings, cells were held at −70 mV and stepwise current injections, 10 mV in magnitude, were applied starting at −60 mV–60 mV for 100 ms. Max conductance (G_max_) was calculated using the following equation: Gmax=ImaxVtest−Vrev, where I_max_ was the maximum capacitance normalized current, V_test_ was the test potential, and V_rev_ was the reversal potential of sodium, which was calculated as 59.8 mV. For experiments using embryonic DRG neurons the calculated residual voltage error was 3.7 ± 0.5 mV, and for adult DRG neurons the calculated residual voltage error was 14.0 ± 2.3 mV. To isolate tetrodotoxin -resistant (TTX-R) currents, neurons were preincubated in standard external solution containing 500 nM TTX (Tocris, cat. no. 1069). To determine the rheobase for each neuron, stepwise current injections were applied in 10 pA increments, starting at −10 pA, for 200 ms until the first action potential was recorded. To determine the action potential profile for each neuron, a single current injection equal to the rheobase of that neuron was applied for 50 ms. To collect excitability data, 2× rheobase for each neuron was applied for 1000 ms. Action potential threshold was determined by calculating when the slope of the first derivative of the membrane potential (dV/dt) reached 20 mV/ms. Action potential half-width was automatically calculated using the Clampex 10.7 software (Molecular Devices, RRID: SCR_011323). SLiPs were added 24 h prior to experimentation. To stimulate PKA activity, neurons were incubated with the cell-permeating cAMP analog 8-Br-cAMP at a final concentration of 250 μM for 30 min at 37°C immediately before recording.

For all voltage-clamp recordings, the following internal solution recipe was used: 130 mM CsCl, 13 mM CsF, 10 mM TEAC, 1 mM MgCl_2_, 1 mM EDTA, and 10 mM HEPES, with a pH of 7.2. For current clamp experiments, the following internal solution recipe was used: 124 mM potassium gluconate, 2 mM MgCl2, 13.2 mM NaCl, 1 mM EGTA, 10 mM HEPES, with a pH of 7.2. For all electrophysiology recordings, the following bath solution recipe was used: 140 mM NaCl, 5.4 mM KCl, 1 mM CaCl2, 1 mM MgCl2, 15.6 mM HEPES, and 10 mM glucose, with a pH of 7.4. All electrophysiology experiments were conducted at RT on a Nikon Eclipse Ti microscope (Nikon) and the data acquired using Multiclamp-700B, sampled at 20 kHz, and filtered at 2 kHz. Data acquisition was monitored and controlled using pClamp 10.7 and analyzed using Clampex 10.7. Traces were generated in OriginPro 8.5 (OriginLab, RRID: SCR_014212).

#### Preparation of microfluidic devices

Microfluidic devices were prepared as previously published.^91^ Briefly, 2-compartment devices (Xona Microfluidics, cat. no. SND150) were used. Glass bottom dishes of 55 mm diameter (Cellvis, cat. no. D60-30-1.5-N) were cleaned and sterilized with 70% ethanol. Device coating was performed with 0.5 mg/mL poly-L-lysine (Sigma-Aldrich, cat. no. P2636), followed by gentle device bonding onto the plates. The assembled devices were coated with 40 μg/mL laminin dissolved in Neurobasal media (Thermo Fisher Scientific, cat. no. 21103049).

#### Calcium imaging

For the mouse eDRG experiments, the eDRG neurons were plated on plastic 35 mm dishes pre-coated with PDL and laminin. eDRG neurons were allowed to mature as stated above until the day of experimentation. 24 h before the experiment, 30 μM of either the Scram or Nav1.8 SLiP was added to the media and incubated with the cells for 24 h at 37°C. The following day, the cells were incubated with 4 μg/mL of Fura2-AM (Thermo Fisher Scientific, cat. no. F1221) for 50 min at room temperature (RT). Cells were subsequently washed with standard extracellular solution (SES) twice, then 2 mL of fresh SES was added to the cells for recording. Live imaging was performed on an inverted Nikon Eclipse Ti microscope with 340- and 380-nm filters controlled by a Ludl Mac6000 shutter controlled with NIS-Elements software (Nikon, RRID: SCR_014329). Fluorescent images were captured every 3 s. Cell treatments were performed using a gravity-based perfusion system. Cells were constantly perfused with SES for 2 min to remove perfusion related artifacts. Then, cells were exposed to freshly prepared KCl (80 mM) for 10 s. Afterward the cells were perfused with SES for 2 min.

For microfluidic experiments, eDRG neurons were unilaterally seeded in a two-chamber microfluidic device (Xona Microfluidics). At 8 DIV SLiPs were added to the axonal compartments and allowed to incubate for 24 h at 37°C. The following day, the somatic compartment was incubated for 1 h with 2 μM of the calcium indicator dye Fluo4-AM (Thermo Fisher Scientific, cat. no. F14217) in imaging buffer consisting of HBSS [Ca^2+^ and Mg^2+^- free] (Thermo Fisher Scientific, cat. no. 14175103) supplemented with 10 mM HEPES, 2 mM CaCl_2_, 1 mM MgCl_2_, 2 mM probenecid (Sigma-Aldrich, cat. no. P8761), pH 7.4 with NaOH. Prior to recording fluorescence activity, DRG cell body compartments were filled with an imaging buffer. Upon recording, the axonal compartment was stimulated with 80 mM KCl and recorded the calcium responses of the DRG cell bodies. Live imaging was performed on a Nikon Eclipse Ti microscope with 340- and 380-nm filters controlled by a Ludl Mac6000 shutter controlled with NIS-Elements software. The regions of interest (ROIs) were drawn around each corresponding soma and fluorescent images were acquired at 510 nm at 1 frame per second (FPS).

#### Zymosan

Mice were brought below a surgical plane of anesthesia with isoflurane (2.5% induction and 1.5% maintenance). After confirming the animal was sufficiently anesthetized by confirming no response to hind paw pinches, a plantar injection of 20 μL of zymosan (Sigma-Aldrich, cat. no. Z4250; 5 mg/mL in saline) was administered into the left hind paw.

#### Spared nerve injury (SNI)

Mice were brought below a surgical plane of anesthesia with isoflurane (2.5% induction and 1.5% maintenance). After confirming the animal was sufficiently anesthetized, characterized by no response to hind paw pinch, the surgical field was prepared. First, hair on the thigh was removed with clippers, then hair-removal cream was used to remove small/fine hairs. Then the area was sterilized with ethanol then iodine. A 3–5 mm skin incision was made in the sterilized area to expose the muscle. Sterilized sticks were used to manipulate the muscle and isolate the sciatic nerves. When the sciatic nerve trifurcation was identified, the tibial and peroneal nerves were tightly ligated with gut suture then transected. Skin sutures were made using 6–0 silk surgical suture (Ethicon, cat. no. K889H). Aseptic techniques were maintained for the duration of surgery.

#### Neuroma

Animals were prepared as previously described.^67^ Briefly, animals were prepared in a similar manner as the SNI surgery with slight modifications. Tight ligatures were tied 1 and 3 mm above the sciatic nerve trifurcation. Then, the sciatic nerve tissue between the ligatures was carefully cut and removed. The nerve was carefully reseated under the muscle, and the incision was sutured closed using 6–0 silk surgical suture. Animals were given 8 weeks to develop neuropathic pain-like behaviors before the start of behavioral assessments. Aseptic techniques were maintained for the duration of surgery.

#### Behavioral assays

For all behavioral assays, animals were habituated to the testing room and testing chamber 2 days before the start of the experiment. This habituation period lasted 2 h total, 1 h in the home cage, and 1 h in the testing chamber. All habituation was conducted in the presence of the experimenter. Habituation and experiments were first conducted on males, then female animals. All chambers were cleaned in between experiments and allowed to dry for 20 min before beginning experiments with a new cohort. For all behavioral assays, the experimenter was blinded.

#### *In vivo* SLiP administration

For SNI and zymosan experiments, aliquots of either the Nav1.8 or Scram SLiP were dissolved in sterile saline and injected into the hind paw ipsilateral to the injury 24 h prior to experimentation. For all neuroma experiments, a silicone cuff was surgically placed around the neuroma bulb as previously described.^92^ After placement, the cuff was then filled with either SLiP. Animals were allowed 24 h to recover from the surgery before beginning experiments.

#### Thermal behavior

Thermal sensitivity was determined using a Hargreaves apparatus (Ugo Basile). After habituation, the Hargreaves apparatus was maneuvered underneath the hind paw of the animal and switched on. The latency to withdrawal was recorded by the experimenter. A 15s cut off was applied to prevent tissue injury. Each hind paw was recorded 3–4 times, with a 5-min break between each trial. The individual trials for each hind paw were averaged and plotted.

#### Cold behavior

Cold sensitivity was determined using acetone evaporation. A 3 mL plastic syringe was filled with acetone, and a small bead of acetone (<0.5 mL) was created at the tip. On an elevated wire mesh platform, the small bead of acetone was placed on the hind paw ipsilateral to the injury, and a timer was started immediately. The time from initial acetone exposure and cessation of behavioral bouts (paw licking, rapid paw withdrawal, paw kicking, etc.) was recorded and plotted.

#### Mechanical behavior

Mechanical sensitivity was determined using Touch Test von Frey filaments. In the zymosan assay, filaments were presented to the plantar surface of the hind paw, and a binary ‘yes’ or ‘no’ response was recorded. This was repeated 5 times, with a 1-min gap between each presentation. The percent response for the presented filament was calculated and plotted.

For withdrawal threshold determination in the SNI model, filaments were presented to the hind paw plantar surface using the Up-Down method. The response pattern was recorded and inputted into Up-Down Reader,^93^ a program that calculates the 50% withdrawal threshold. The data was then plotted.

For quantification of nocifensive response probability in the neuroma model, transcutaneous neuroma poke was conducted as previously described.^67^ Each animal was tested a total of 10 trials, and limb flicking responses to each stimulation were recorded and used to calculate response probability.

#### Spontaneous behavior

Spontaneous pain-like behaviors in the neuroma model were measured using a previously described home-cage EMG/video recording system.^67^ Briefly, wireless telemetry transmitters were implanted into the vastus lateralis muscle ipsilateral to the injury, and mice were allowed to recover before sciatic nerve transection. Animals were singly housed in home cages equipped with overhead night-vision cameras, and simultaneous video and EMG signals were recorded over 24 h. Raw EMG signals were bandpass filtered at 5–30 Hz, and limb flicks were identified as bouts of repetitive EMG spikes. Candidate events were first detected using EMG readouts then manually verified against synchronized video recordings by a blinded experimenter.

#### *In vivo* DRG imaging in response to neuroma poke

5 days post SLiP administration (silicone cuff implant), the imaging experiment was performed as described previously.^67^ Briefly, adult Vglut2-ires-cre knock-in (C57BL/6J) mice (The Jackson Laboratory, RRID: IMSR_JAX:028863) were crossed with Ai95(RCL-GCaMP6f)-D (C57BL/6J) (The Jackson Laboratory, RRID: IMSR_JAX:028865) mice to generate Vglut2-ires-Cre;Ai95D mice that express the fluorescent calcium sensor GCaMP6f in excitatory neurons. Then, adult Vglut-ires-Cre;Ai95D mice (8–12 weeks post sciatic nerve transection) were anesthetized with isoflurane (2.5% induction, 2% maintenance) and kept at 37°C. After shaving the back, a dorsal laminectomy was performed to expose the L4 DRG. Bleeding was controlled with absorbent paper points, and a custom spinal clamp was used to minimize motion. The DRG was illuminated with 470 nm LED light through a 10×/0.3 NA objective. The neuroma was then surgically exposed, and mechanical stimuli using 1.4 g von Frey filament were applied to the neuroma. Imaging parameters were 10 Hz acquisition and 99 ms exposure. ROIs were manually identified and were included only if ΔF/F exceeding 3× baseline noise for ≥0.5 s.

#### Locomotor activity measurement

A previously described behavioral recording system^94^ was used for assessing locomotor activity before and after drug treatments. Briefly, mice were placed in a black acrylic enclosure (18 × 18 × 15 cm; length × width × height) that was open only at the bottom. The enclosure was positioned on a 25-cm square, 5-mm-thick glass platform. Two independent near-infrared (NIR) LED strips (850 nm; SMD5050-300-IR, Huake Light Electronics Co., Ltd., Shenzhen, China) were mounted 10 cm beneath the glass floor and powered by a 12 V DC supply. Recordings were acquired in darkness using a Basler acA2000-50gmNIR GigE NIR-sensitive camera (Basler AG, cat. no. 105713, Ahrensburg, Germany) positioned 30 cm below the glass surface. Video acquisition was performed with Pylon Viewer software (v4.1.0.3660, Basler) under standardized camera settings. All videos were recorded at 25 frames per second with a resolution of 500 × 500 pixels. For each recording, the tail-base position was extracted from body frames using DeepLabCut (RRID: SCR_021391). Frame-to-frame movement was calculated as the Euclidean distance between tail-base positions in consecutive frames. Cumulative locomotion over time, expressed as pixel distance, was defined as the sum of all frame-to-frame distances.

#### Immunoprecipitation

Dissected mouse neuronal tissue, DRGs and sciatic nerves, were homogenized in cold NP-40 lysis buffer (Thermo Fisher Scientific, cat. no. J60766.AP) containing protease inhibitors (Sigma-Aldrich, cat. no.1183617000). Protein concentration was determined using a Pierce BCA protein assay kit (Thermo Fisher Scientific, cat. no. 23227). Immunoprecipitation was then conducted using Protein A/G magnetic beads (Selleck Chemical, cat. no. B23201) following manufacturer’s instructions with no modifications. For co-immunoprecipitation in the presence of SLiPs, the SLiPs were added to the lysate and incubated for 1 h at RT before antibody addition. Following immunoprecipitations, samples were separated on a 3–8% gradient Tris-acetate gel (Thermo Fisher Scientific, cat. no. EA03755BOX) and then transferred to nitrocellulose membranes using the Turbo Trans Blot system (Bio-Rad, cat. no. 1704158). Membranes were then blocked with 5% dry milk in TBST overnight at 4°C. After blocking, membranes were probed using the appropriate primary antibody: Ank1 (Rabbit anti-Ank1, Proteintech, cat. no. 23938-1-AP, RRID: AB_3085717; 1:2000), Ank2 (Rabbit anti-Ank2, Bioss, cat.no. BS-6967R; 1:2000), or Ank3 (Rabbit anti-Ank3; 1:2000 or Mouse-Anti-Ank3; 1:2000) overnight at 4°C. Secondary antibody incubation was conducted for 2 h at room temperature (Donkey anti-rabbit IRDye-680RD, LICORBio cat.no. 926–68073, RRID: AB_10954442; 1:10000 or Goat anti-mouse IRDye-800CW, LICORBio cat.no. 827–08364, RRID: AB_10793856; 1:10000). Images were taken on Bio-Rad ChemiDoc imaging system.

#### Proteomic analysis

Animals were euthanized with CO_2_ and were dissected to bilaterally collect uninjured sciatic nerves and injured neuromas at 8–10 weeks post sciatic nerve transection. Pain intensity was phenotyped before tissue collection. Neuromas from mice that exhibited limb flicking responses to transcutaneous neuroma poke with a probability ≥0.7 were defined as high-pain neuromas, as previously described.^67^ Samples were flash frozen with dry ice and sent to the UCSF Mass Spectrometry Facility for analysis. Protein quality and subsequent analysis was conducted as previously described.^95^ Details are provided below.

#### Protein extraction and in solution digestion

Samples were thawed and homogenized in 100 μL 8 M Guanidine Hydrochloride (GndHCl), using a probe sonicator (Thermo Fisher Scientific). Insoluble material was removed by centrifugation at 14000 × g for 10 min. Protein concentration in the extracts was measured using the Pierce BCA Protein Assay Kit in triplicates, taking 1 μL aliquots of this solution. Aliquots of samples containing 150 μg of protein were added 9× the sample volume of ethanol and allowed to precipitate at −80C overnight. After that, pellets were centrifuged at 15000 rcf for 10 min, the supernatant was removed, and the samples were allowed to dry. The pellets were resuspended in 6.25 μL of resuspension buffer containing 6.25 M guanidine hydrochloride, 6.25% V/V of Sigma-Aldrich Phosphatase inhibitors cocktails 2 and 3, 78 mM triethylammonium bicarbonate (TEAB) pH 8.0, 7.8 mM Tris(2-carboxyethyl)phosphine hydrochloride and 1% (W/V) zwitterionic 3–16 detergent (Sigma-Aldrich, cat. no. 693023). Samples were sonicated in a bath sonicator for 5 min, then incubated at 56°C for 30 min, followed by a 30-min incubation at room temperature in the dark with 20 mM iodoacetamide. The samples were added 31.3 μL 100mM TEAB containing 1.3 μg Lysyl endopeptidase (LysC) (FUJIFILM-Wako, cat. no. 125–05061), and incubated at 37C o/n. After that, the samples were added 5% (W/W) modified trypsin (TPCK-Trypsin, Thermo Fisher Scientific, cat. no. 20233) and incubated overnight at 37°C.

Digested Samples were labeled according to TMTProTM-18 label plex kit instructions (Thermo Fisher Scientific, cat. no. A52045), with some modifications. Briefly, TMT reagents were dissolved in acetonitrile at 25 mg/μL, and 10 μL of these stocks added to the samples (250 μg reagent, 1.7-fold the peptide mass amount). After incubation for 1 h at room temperature samples were quenched with 1 μL 5% hydroxylamine, then all samples were combined and added over 20 mL 0.1% formic acid and desalted using a C18 SepPak cartridge (Waters, cat. no. wat054955). The Sep Pak eluate was dried in preparation for phosphopeptide enrichment.

#### Enrichment of phosphorylated peptides using titanium dioxide

Phosphopeptide enrichment was performed in an AKTA Purifier (GE Healthcare, Piscataway, NJ) using 5 μm titanium dioxide (TiO2) beads (GL Sciences, cat. no. 5020–75000, Tokyo, Japan) in-house packed into a 4.0 mm × 3 cm analytical column (Upchurch Scientific, Oak Harbor, WA). Combined TMT labeled tryptic digests were resuspended in 240 μL buffer containing 35% MeCN, 200 mM NaCl, 0.4% TFA and loaded onto the TiO_2_ column at a flow rate of 2 mL/min. The non adsorbed peptides (non-phosphorylated material, flow though fraction) were recovered. The column was then washed for 2 min with 35% MeCN, 200 mM NaCl, 0.4% TFA to remove non phosphorylated peptides. Phosphopeptides were eluted from the column using 1 M potassium phosphate monobasic (KH_2_PO_4_) at a flow rate of 0.5 mL/min for 30 min, directly onto an on-line coupled C18 column (Luna 10 μm C18(2) 100 Å, LC Column 30 × 4.6 mm, Phenomenex) This column was washed with 0.1% trifluoroacetic acid (TFA) for 14 min, and the adsorbed material was eluted in 400 μL of 50% MeCN, 0.1% TFA at a flow rate of 0.25 mL/min. The eluate was solvent evaporated and then resuspended in 240 μL 20 mM ammonium formate pH 10.4 for fractionation of the peptide mixture by high pH RP chromatography. The flowthrough fraction was solvent evaporated, desalted in a C18 SepPack cartridge, then resuspended in 240 μL 20 mM ammonium formate pH 10.4 for fractionation of the peptide mixture by high pH RP chromatography.

#### High pH Reverse Phase chromatography

The phosphopeptide enriched sample and the flow through obtained from the TiO2 chromatography were separately fractionated on an AKTA purifier system utilizing a Phenomenex Gemini 5u C18 110A 150 × 4.60 mm column, operating at a flow rate of 0.550 mL/min. Buffer A consisted of 20 mM ammonium formate (pH 10) and buffer B consisted of 20 mM ammonium formate in 90% acetonitrile (pH 10). Gradient details were as follows: 1% to 30% B in 49 min, 30% B to 70% B in 4 min, 70% B down to 1% B in 4 min. Peptide-containing fractions were collected, evaporated and resuspended in 0.1% formic acid. For phosphopeptides, early and late chromatographic fractions were combined to produce 51 samples for LCMSMS analysis.

#### Mass spectrometry analysis

Aliquots (containing around 3 μg of digested material) coming from Reverse Phase fractionation were run onto a 2 mm, 75mm ID x 50 cm PepMap RSLC C18 EasySpray column (Thermo Fisher Scientific). 3-h MeCN gradients (2–25% in 0.1% formic acid) were used to separate peptides, at a flow rate of 200 nL/min, for analysis in a Orbitrap Lumos Fusion (Thermo Fisher Scientific) in positive ion mode with the following settings. MS spectra were acquired between 375 and 1500 m/z with a resolution of 120000. For each MS spectrum, multiply charged ions over the selected threshold (2E4) were selected for MSMS in cycles of 3 s with an isolation window of 0.7 m/z. Precursor ions were fragmented by HCD using stepped relative collision energies of 30, 35 and 45 to ensure efficient generation of sequence ions as well as TMT reporter ions. MSMS spectra were acquired in centroid mode with resolution 50000 from m/z = 110 in the Lumos. A dynamic exclusion window was applied which prevented the same m/z from being selected for 30s after its acquisition.

#### Peptide and protein identification and TMT quantitation

Peak lists were generated using PAVA in-house software.^96^ All generated peak lists were searched against the human subset of the SwissProt database (SwissProt.2019.07.31), using Protein Prospector^97^ with the following parameters: Enzyme specificity was set as Trypsin, and up to 2 missed cleavages per peptide were allowed. Carbamidomethylation of cysteine residues, and TMTPro16plex labeling of lysine residues and N-terminus of the protein were allowed as fixed modifications. N-acetylation of the N-terminus of the protein, loss of protein N-terminal methionine, pyroglutamate formation from peptide N-terminal glutamines, oxidation of methionine and phosphorylation on serine, threonine and tyrosine were allowed as variable modifications. Mass tolerance was 5 ppm in MS and 30 ppm in MS/MS. The false discovery rate was estimated by searching the data using a concatenated database which contains the original SwissProt database, as well as a version of each original entry where the sequence has been randomized. A 1% FDR was permitted at the protein and peptide level, for the sets of unphosphorylated, monophosphorylated and doubly phosphorylated peptides. For quantitation only unique peptides were considered; peptides common to several proteins were not used for quantitative analysis. Relative quantization of peptide abundance was performed via calculation of the intensity of reporter ions corresponding to the different TMT labels, present in MS/MS spectra. Intensities were determined by Protein Prospector. Summed intensity per sample on each TMT channel for all identified spectra were used to normalize individual intensity values. Relative abundances were calculated as ratios vs. the average intensity levels in the channels corresponding to the relevant control samples. Spectra representing replicate measurements of the same peptide were kept. Individual spectral ratios were aggregated to the peptide level using median values of the log2 ratios. Non phosphorylated peptides were used to estimate relative protein levels. For total protein relative levels, peptide ratios were aggregated to the protein levels using median values of the log2 ratios. Phosphopeptide ratios were corrected by the available protein ratios. Statistical significance was calculated at the phosphopeptide and protein levels by comparing the values for the 3 biological replicates of the relevant groups in the TMT experiment with a 2-tailed *t* test.

### Quantification and statistical analysis

All statistical tests were performed using Prism 10.5 (GraphPad, RRID: SCR_002798). Normality of data distribution for all data was tested using Kolmogorov-Smirnov, D’Agostino & Pearson, Anderson-Darling, and Shapiro-Wilk tests. Any dataset that did not pass at least three out of four tests of normality underwent the appropriate non-parametric statistical analysis. All data are shown as means ± S.E.M. For behavioral experiments, power analysis was conducted to achieve detection limits with a α-value set at 0.05. Statistical significance was determined utilizing a *p*-value <0.05 for all experiments. For data presented without a *p*-value, the following symbols are used to represent significance: ∗*p* < 0.05; ∗∗*p* < 0.01; ∗∗∗*p* < 0.005; ∗∗∗∗*p* < 0.0001. Repeated measure two-way ANOVA statistical tests with multiple comparisons and an appropriate post-hoc correction, one-way ANOVA with Holms-Sidak correction, Kruskal-Wallis, or Student’s *t* test were used where appropriate. Additional statistical information can be found in the figure legends accompanying each figure.

## Supplemental information

Document S1. Figures S1–S11

Document S2. Article plus supplemental information
