# Supplementary material for: Disruption of the Nav1.8/Ankyrin-3 interaction in nociceptors reduces pathological pain-like behaviors in mice
Source: Cell Rep Med. 2026 Oct 6;7(10):103090. doi: 10.1016/j.xcrm.2026.103090 (PMC13710374; doi:10.1016/j.xcrm.2026.103090)
Supplement: Supplementary file 1 [file mmc1.pdf]

Cell Reports Medicine, Volume *7*

Supplemental information

Disruption of the Nav1.8/Ankyrin-3 interaction in nociceptors reduces pathological pain-like behaviors in mice

Rasheen Powell, Xiangsunze Zeng, Bruna Lenfers Turnes, Hyoung Woo Kim, Veselina Petrova, Selwyn S. Jayakar, Emily Shea, Keunjung Heo, Emma Cropper, Maryam Arab, Georgios Kimourtzis, Juan A. Oses-Prieto, Alma L. Burlingame, Stephen G. Waxman, Sulayman D. Dib-Hajj, and Clifford J. Woolf

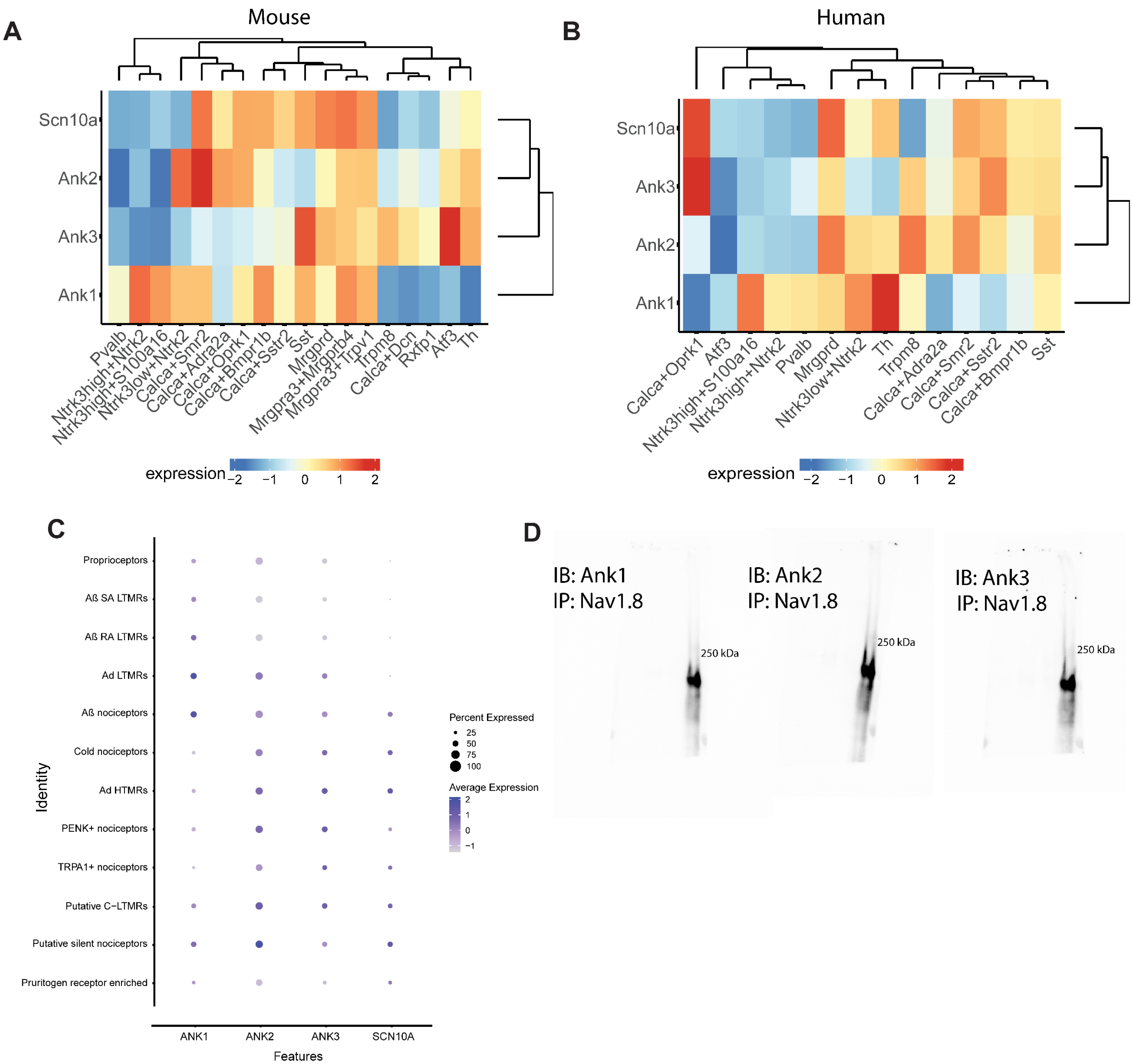

<u>Figure S1. Nav1.8 and ankyrin isoforms are co-expressed in sensory neuron subsets in mice and in humans. Related to Figures 1 and 2.</u> (A) Heatmap displaying transcriptomic expression of ankyrin isoforms and Nav1.8 in mouse sensory neurons. (B) Heatmap displaying transcriptomic expression of ankyrin isoforms and Nav1.8 in humans. (C) Dot plot Heatmap displaying transcriptomic expression of ankyrin isoforms and Nav1.8 in human DRGs. (D) Uncropped western blot images of IP pull down of Nav1.8 from mouse DRGs and sciatic nerves. Nav1.8 was pulled down from lysates and ankyrin isoforms were immunoblotted. Data in (A) and (B) was sourced from Painseq.io. Data displayed in (C) was sourced from sensoryomics.shinyapps.io.

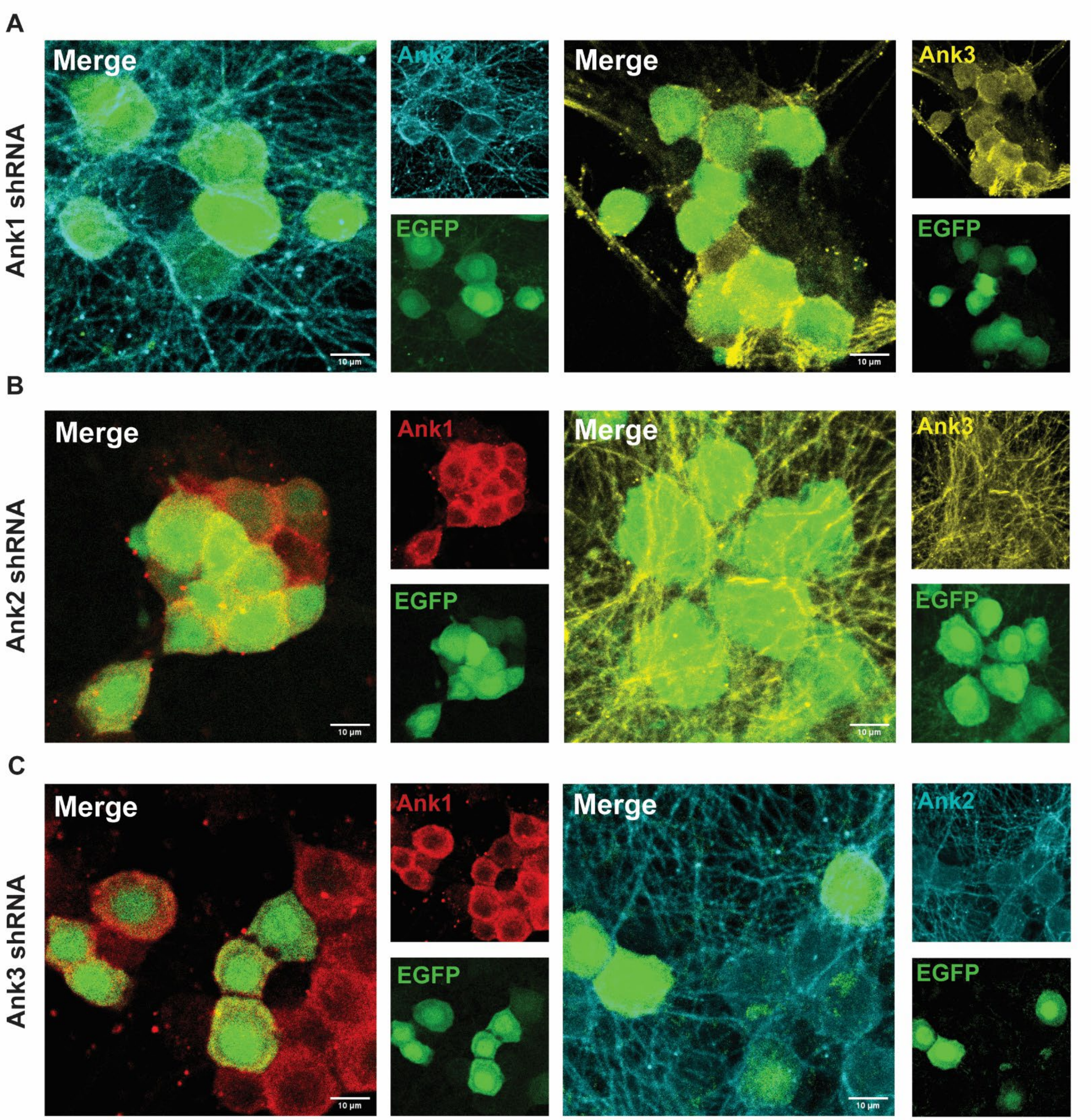

Figure S2. Ankyrin shRNA knockdown is specific for the targeted ankyrin isoform. Related to Figure 2. (A) Representative immunofluorescent images of cells transduced with AAV-Ank1shRNA-EGFP. [Left] Immunostaining for Ank2. [Right] Immunostaining for Ank3. (B) Representative immunofluorescent images of cells transduced with AAV-Ank2shRNA-EGFP. [Left] Immunostaining for Ank1. [Right] Immunostaining for Ank3. (C) Representative immunofluorescent images of cells transduced with AAV-Ank3shRNA-EGFP. [Left] Immunostaining for Ank1. [Right] Immunostaining for Ank2. In all cases, there was no significant loss in immunoreactivity for the off-target ankyrin isoforms. All scale bars are 10 µm in length.

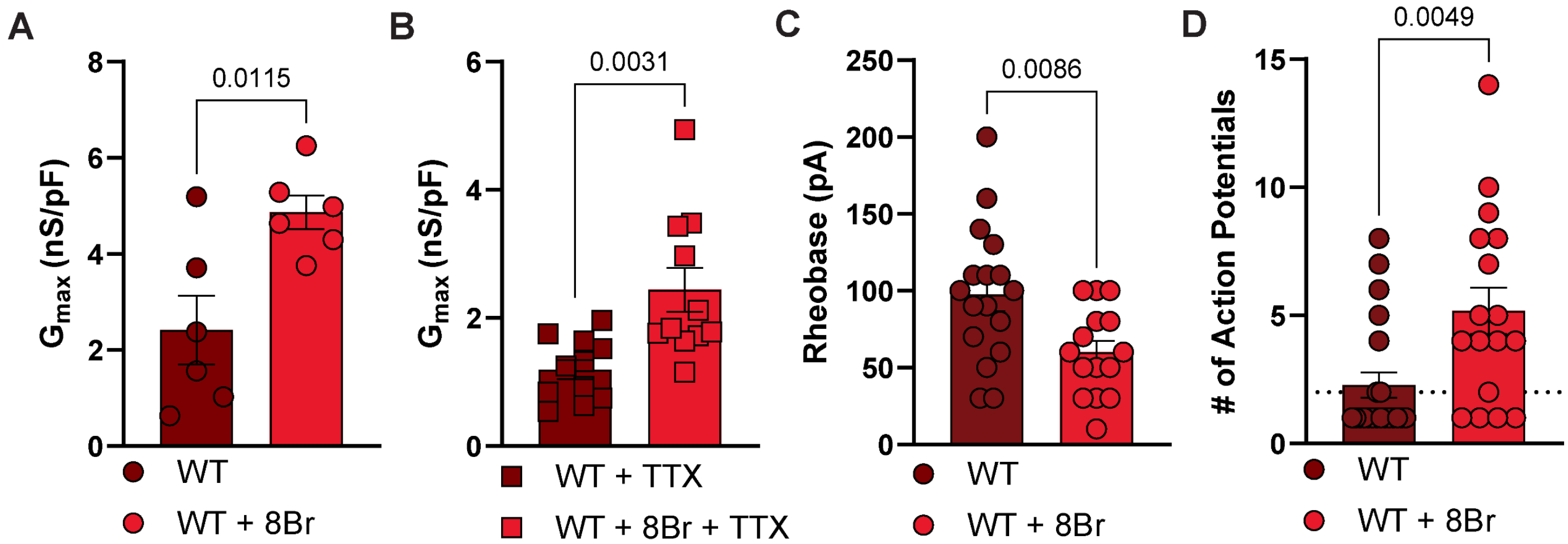

<u>Figure S3. 8-Br-cAMP increases sodium channel conductance in cultured eDRG neurons driving metrics of excitability. Related to Figures 2 and 3.</u> (A) Total sodium channel conductance of wild type eDRG neurons (WT; n = 6, WT + 8-Br-cAMP; n = 6). (B) TTX-R sodium channel conductance of wild type eDRG neurons (WT; n = 11, WT + 8-Br-cAMP; n = 11). (C) Rheobase of eDRG neurons with (n = 15) and without 8-Br-cAMP (n = 17). (C) Total number of action potentials elicited following a suprathreshold stimulus (2x rheobase) for 1 second (WT; n = 21, WT + 8-Br-cAMP; n = 17). Statistical significance determined by paired students t-test. All data presented as mean ± s.e.m.

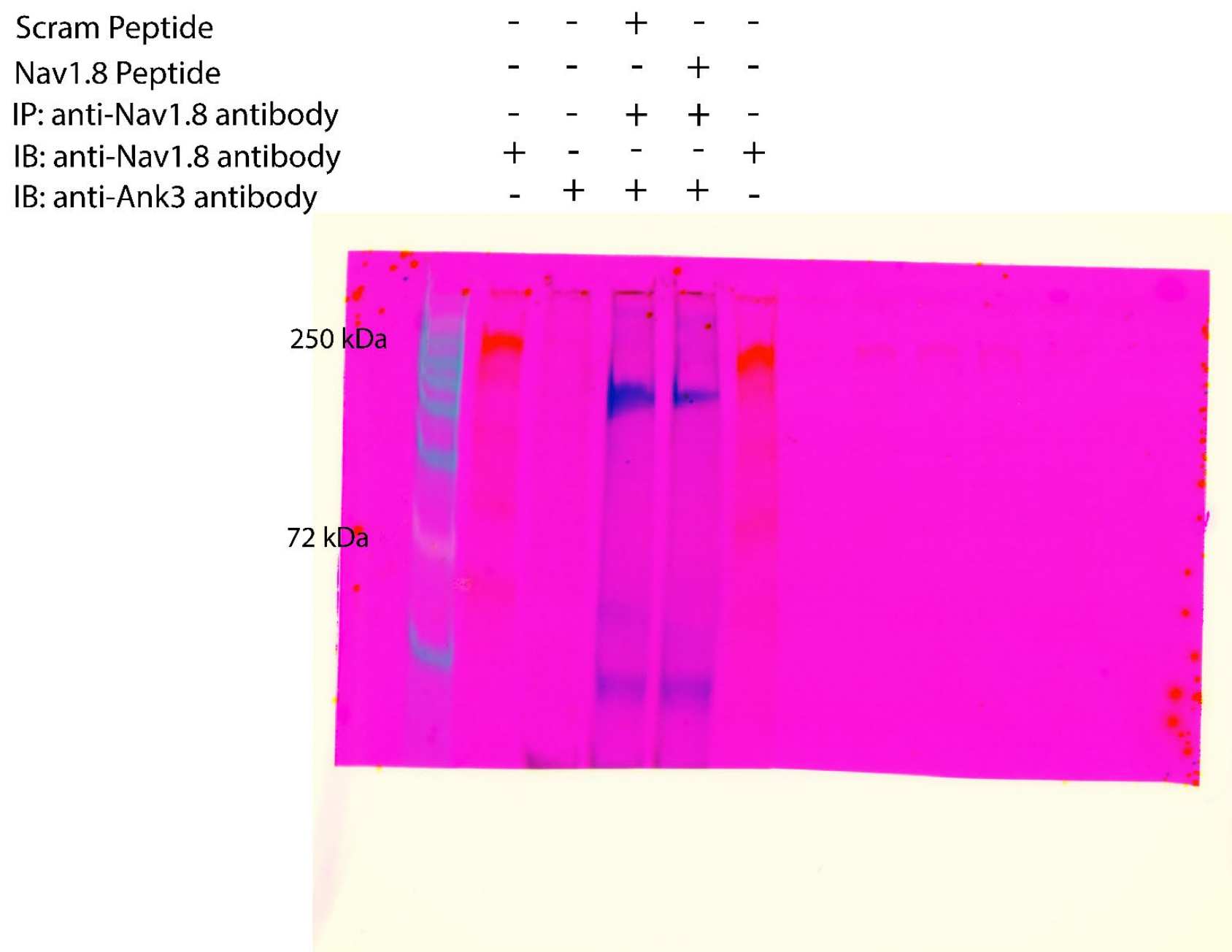

<u>Figure S4. Nav1.8 SLiP reduces Nav1.8 pulldown of Ank3 in mouse DRGs and sciatic nerves. Related to Figures 3, 4, 5, and 6.</u>
Uncropped blot of Nav1.8 pulldown of Ank3 in co-immunoprecipitation assay. Lane 1 is the molecular ladder. Lane 2 is the input. Lane 3 is the no-Nav1.8-antibody control. Lane 4 is the Co-IP in the presence of Scrambled peptide (Scram SLiP without myristoyl group). Lane 5 is the Co-IP in the presence of the Nav1.8 peptide (Nav1.8 SLiP without myristoyl group). Lane 6 is a replicate of Lane 2.

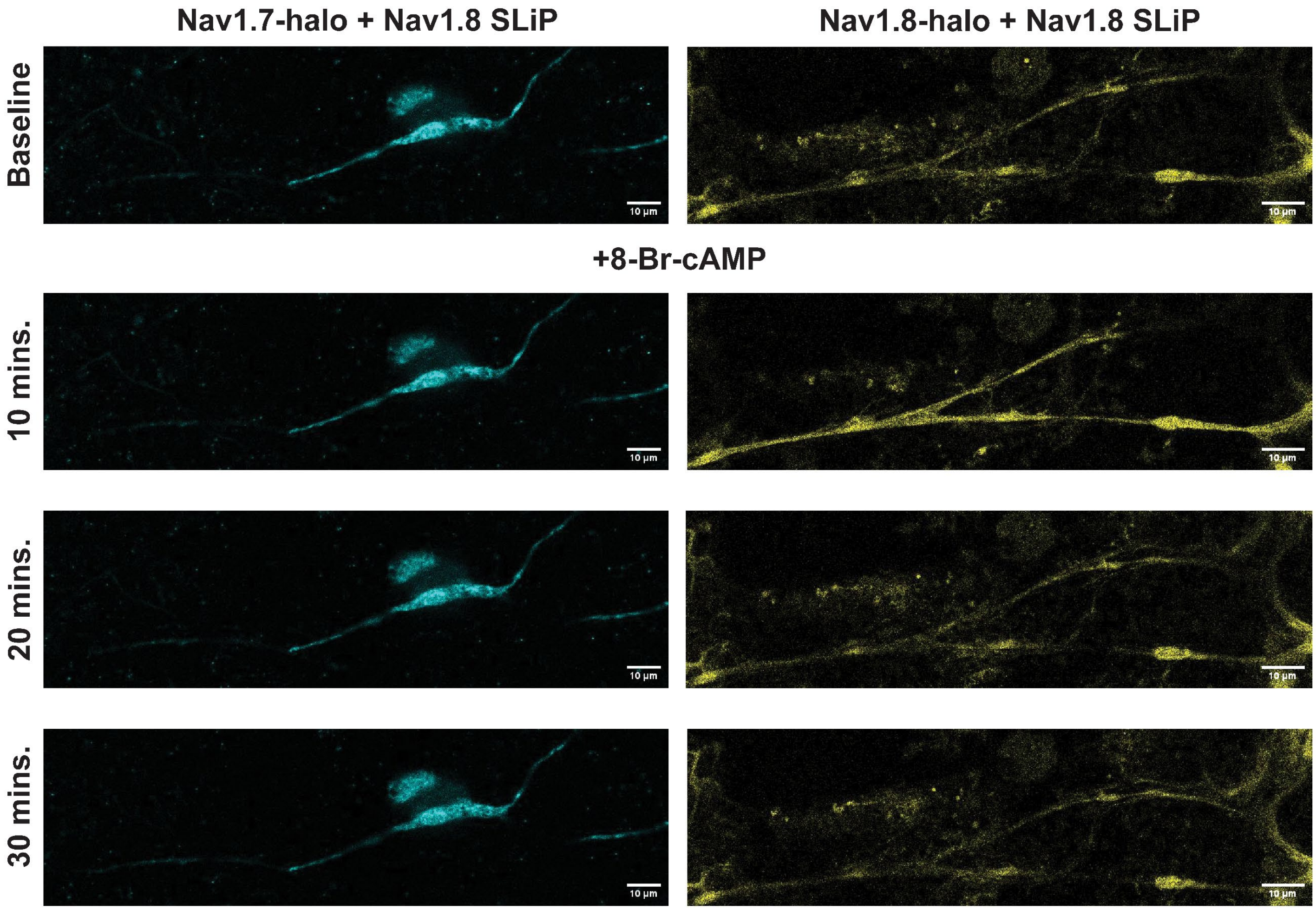

<u>Figure S5. Nav1.8 SLiP does not affect Nav1.7 membrane expression following cAMP kinase activation with 8-Br-cAMP. Related to Figure 3 and 4.</u> Representative immunofluorescent images showing membrane expression of either [Left] halo-tagged Nav1.7 or [Right] halo-tagged Nav1.8. Live-cell imaging was conducted using a cell impermeable halo-tag ligand to track membrane expression following 8-Br-cAMP exposure. Only Nav1.8-halo displayed a decrease in fluorescent signal in the presence of the Nav1.8 SLiP. All scale bars are 10 µm in length.

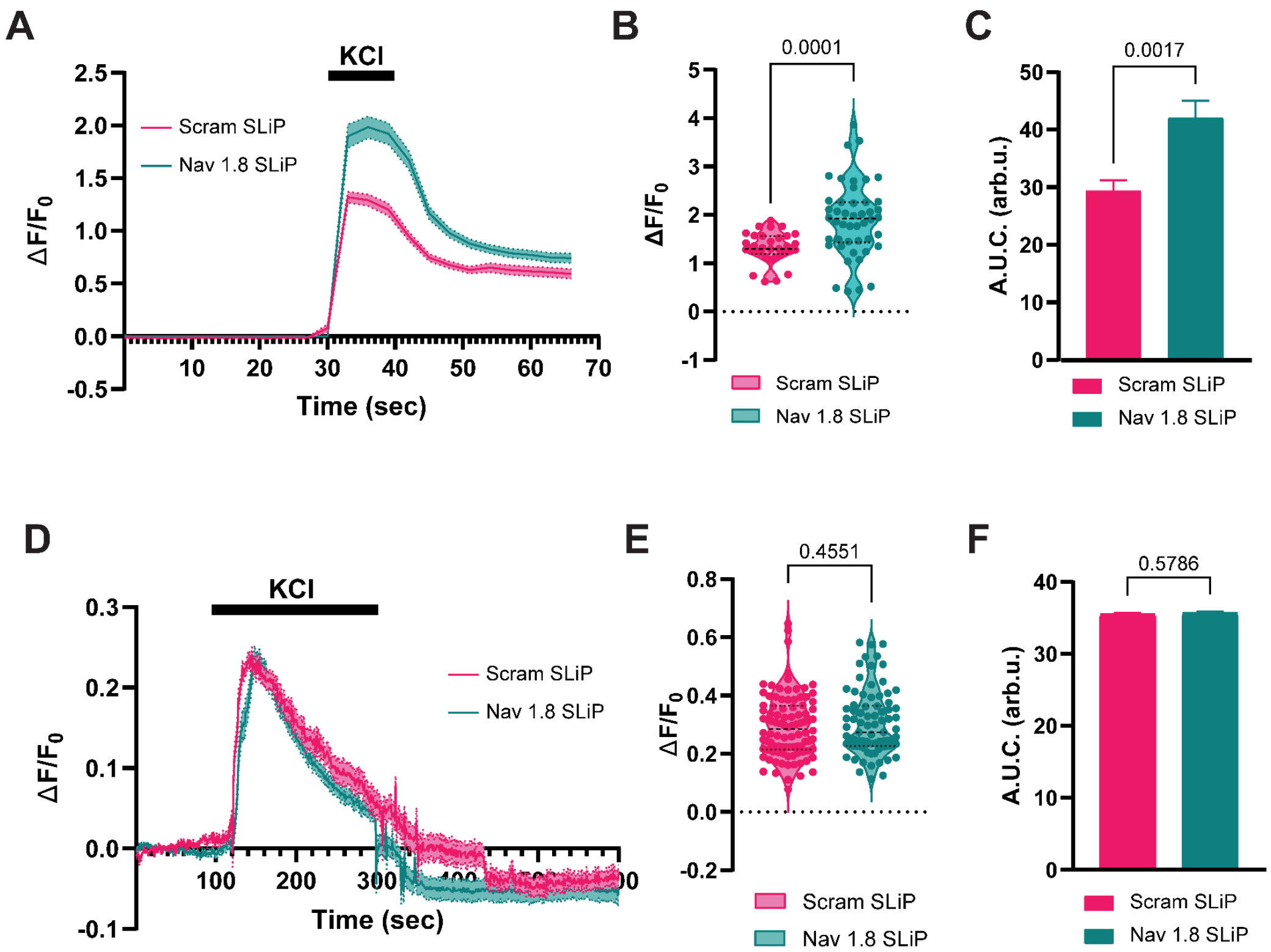

Figure S6. Nav1.8 SLiP effect on calcium flux in basal conditions. Related to Figure 5. (A – C) Calcium imagining experiments where SLiPs were added to the entire cell (bath incubation) in a basal/physiological condition. (A) Averaged change in fluorescence for the Scram (n = 33) and Nav1.8 (n = 46) SLiP groups following depolarization with KCl. (B) Peak change in fluorescence of curves in (A). (C) Area under curves presented in (A). (D – F) Calcium imaging experiments where SLiPs were added onto the axons in basal/physiological conditions: (D) Averaged change in fluorescence for the Scram (n = 96) and Nav1.8 (n = 81) SLiP groups following axonal depolarization with KCl. (E) Peak change in fluorescence in curves presented in (D). (F) Area under the curves presented in (D). For (B), (C), (E), and (F) significance was determined using a two-tailed unpaired students t-test. All data presented as mean ± s.e.m.

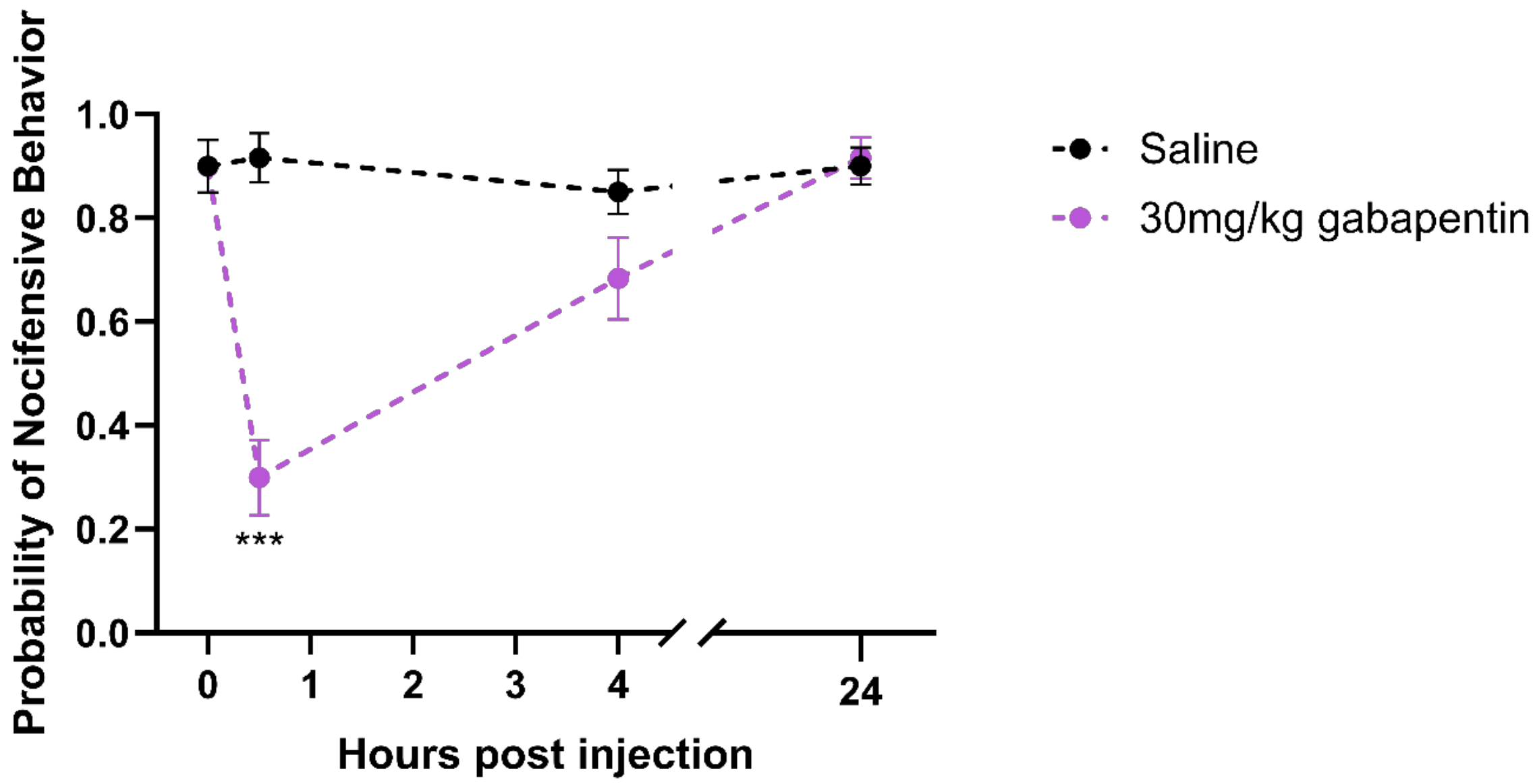

Figure S7. Efficacy of gabapentin in neuroma model. Related to Figure 6. Mice that developed prototypical nocifensive behavior (limb flick) to transcutaneous neuroma mechnical poke were IP administered either saline (n = 6; 4 males and 2 females) or 30 mg/kg gabapentin (n = 6; 4 males and 2 females). Response to the transcutaneous neuroma poke was tested at the indicated time points. Animals that received gabapentin showed peak analgesic efficacy 30 minutes after injection. Statistical significance was determined using two-way ANOVA with multiple comparisons and Holm-Šídák post-hoc correction. Asterisks represent the following significance thresholds: *p < 0.05; **p < 0.01; ***p < 0.005; ****p < 0.0001. Data presented as mean ± s.e.m.

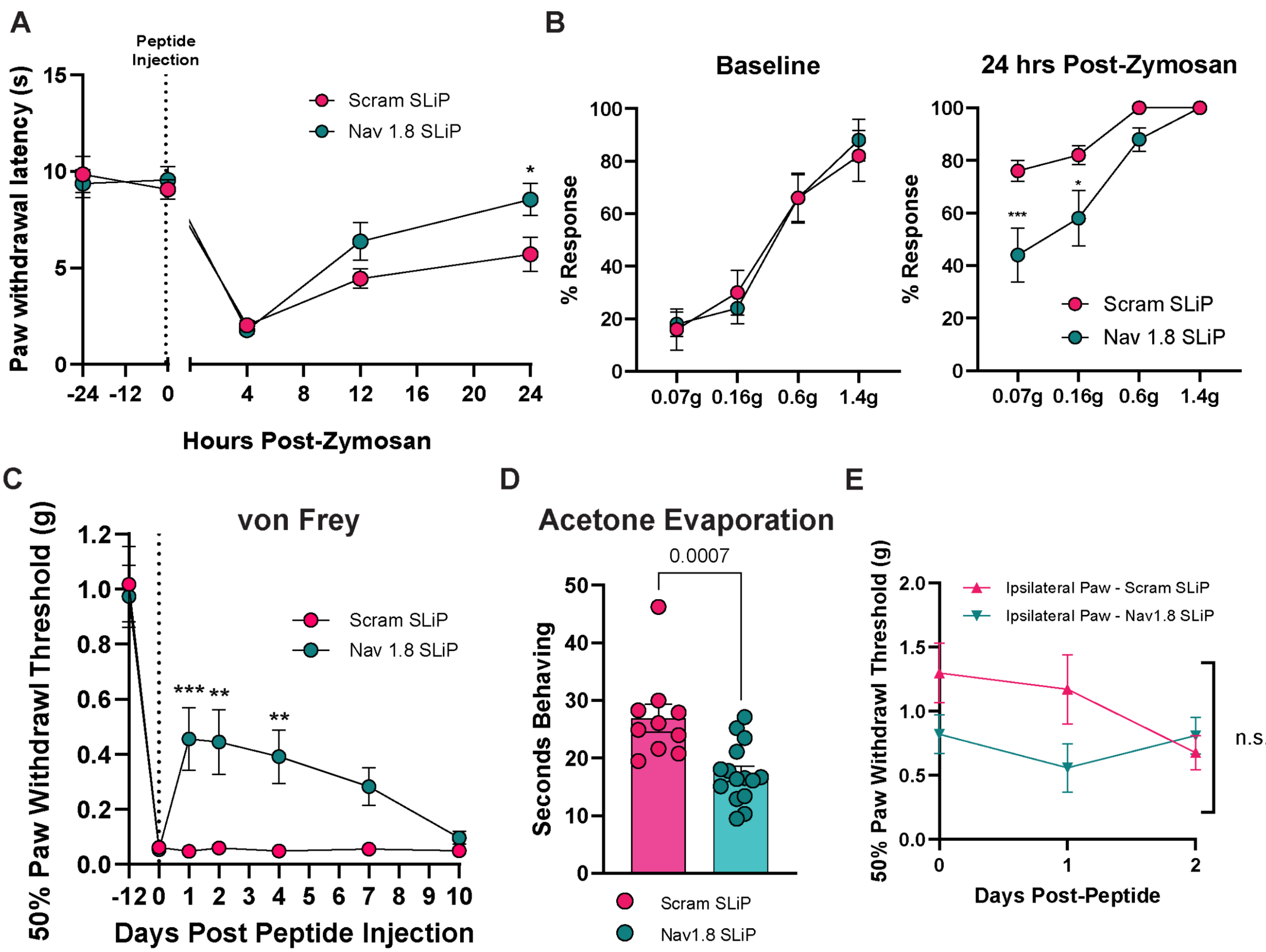

<u>Figure S8. Nav1.8 SLiP reduces stimulus-evoked pain measures in models of acute inflammatory and neuropathic pain but not acute nociceptive pain. Related to Figure 6.</u> (A) Hargreaves assay to test thermal sensitivity of animals before and after zymosan injection. SLiPs were injected into the ipsilateral hind paw 24 hours before zymosan injection (Scram SLiP; n = 10 [5 male and 5 female], Nav1.8 SLiP; n = 10 [5 male and 5 female]). Statistical significance determined by two-way ANOVA with multiple comparisons and Bonferroni post-hoc correction. Nav1.8 SLiP produced no change pre-zymosan. (B) Mechanical sensitivity of mice before and 24 hours after zymosan injection. Animals were injected with either SLiP into the ipsilateral hind paw 24 hours before zymosan injection (Scram SLiP; n = 10 [5 male and 5 female], Nav1.8 SLiP; n = 10 [5 male and 5 female]). Nav1.8 SLiP produced no change pre-zymosan. Statistical significance determined by two-way ANOVA with multiple comparisons and Bonferroni post-hoc correction. (C) Efficacy of Nav1.8 SLiP in spared nerve injury neuropathic pain model. Data presented as 50% paw withdrawal threshold. Animals were injected with SLiP immediately after day 0 behavior. (Scram SLiP; n = 12 [6 male and 6 female], Nav1.8 SLiP; n = 12 [6 male and 6 female]). Statistical significance determined by two-way ANOVA with multiple comparisons and Bonferroni post-hoc correction. *$p < 0.05$; **$p < 0.01$; ***$p < 0.005$; ****$p < 0.0001$. (D) Acetone evaporation cold allodynia test in SNI model. Data presented is the total time mice displayed nocifensive behavior after a single drop of acetone was placed on the plantar surface of the hind paw (Scram SLiP; n = 10 [5 male and 5 female], Nav1.8 SLiP; n = 15 [10 male and 5 female]). Statistical significance determined by unpaired students t-test. (E) Efficacy of Nav1.8 SLiP in uninjured mice. Data presented as 50% paw withdrawal threshold. Animals were injected with SLiP immediately after day 0 behavior (Scram SLiP ; n = 4 [2 male and 2 female], Nav1.8 SLiP; n = 4 [2 male and 2 female]). Statistical significance was determined using two-way ANOVA with multiple comparisons and Holm-Šidák post-hoc correction.

A

```
mNav1.1 TVTVPIAVGESDFENLNTEDFSSESD rNav1.1 TVTVPIAVGESDFENLNTEDFSSESD hNav1.1 TVTVPIAVGESDFENLNTEDFSSESD
mNav1.2 TVTVPIAVGESDFENLNTEEFSSESD rNav1.2 TVTVPIAVGESDFENLNTEEFSSESD hNav1.2 TVTVPIAVGESDFENLNTEEFSSESD
mNav1.3 TVTVPIAVGESDFENLNTEEFSSESE rNav1.3 TVTVPIAVGESDFENLNTEEFSSESE hNav1.3 TVTVPIAVGESDFENLNTEEFSSESE
mNav1.4 TIHVPIASEESDLEMPTEEETDTFSE rNav1.4 TIQVPIASEESDLEMPTEEETDAFSE hNav1.4 TIQVPIASEESDLEMPTEEETDTFSE
mNav1.5 SETVPIAVAESDTDDQEEDEENSLGT rNav1.5 PVCVPIAVAESDTEDQEEDEENSLGT hNav1.5 PVCVPIAVAESDTDDQEEDEENSLGT
mNav1.6 TVRVPIAVGESDFENLNTEDVSSESD rNav1.6 TVRVPIAVGESDFENLNTEDVSSESD hNav1.6 TVRVPIAVGESDFENLNTEDVSSESD
mNav1.7 TVTVPIAPGESDLENMNTEELSSDSD rNav1.7 TVTVPIAPGESDLEIMNTEELSSDSD hNav1.7 TVTVPIAPGESDLENMNAEELSSDSD
mNav1.8 WVSVPIAEGESDLDELEEDVEHASQS rNav1.8 WVSVPIAEGESDLDELEEDMEQASQS hNav1.8 WVSVPIAEGESDLDDLEDDGGEDAQS
mNav1.9 APLAPLAKEEDDMECCGECDASPTSQ rNav1.9 APLAPLAEVEDDVEYCGEGGALPTSQ hNav1.9 TWLAPLAEEEDDVEFSSGEDNAQRIT
```

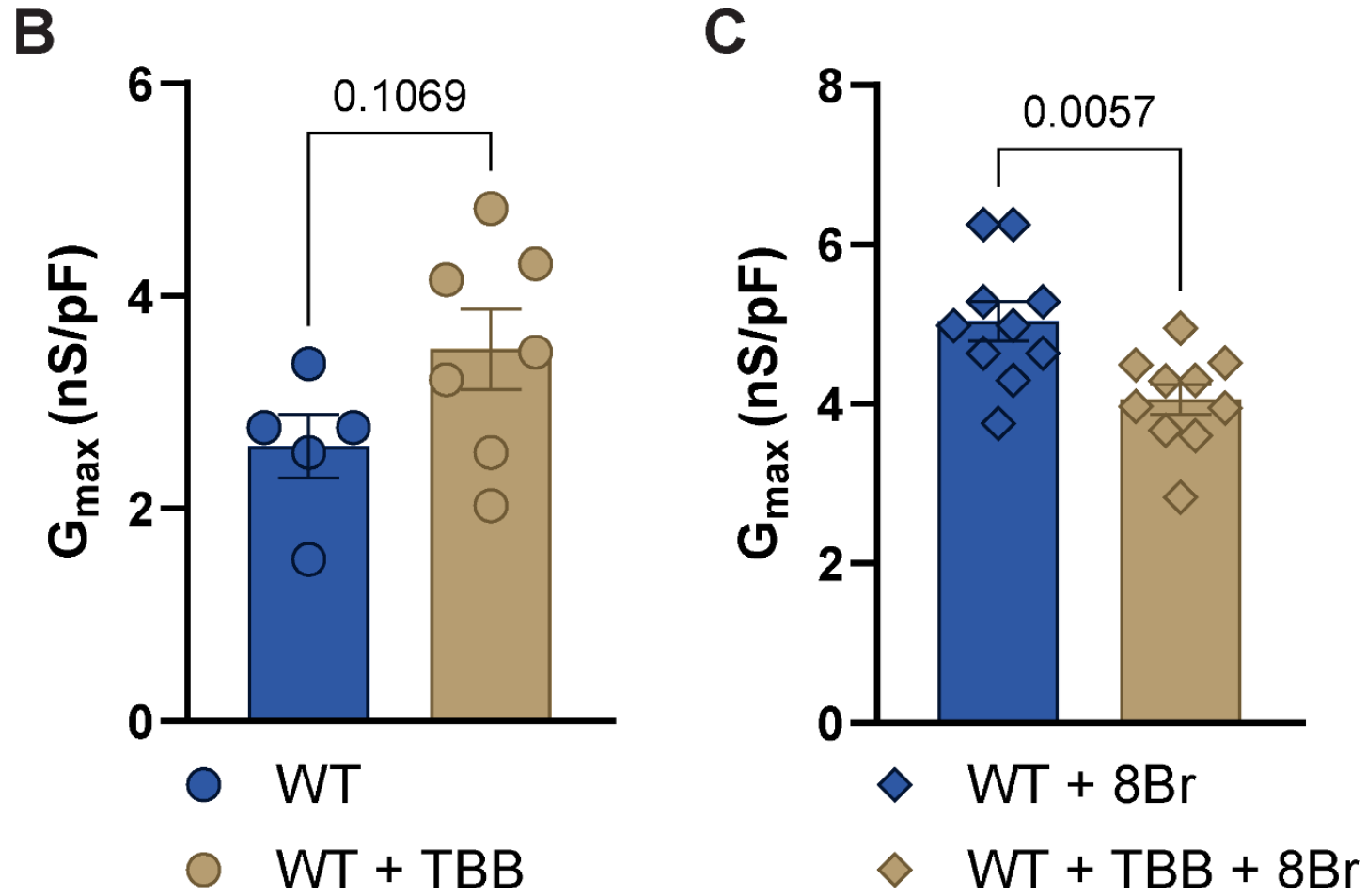

Figure S9. CK2 inhibition reduces total sodium current density in cultured eDRG neurons, however, Nav1.8 lacks canonical CK2 phosphorylation motifs. Related to Figure 3. (A) Sequence alignment of the extended ABD for each Nav channel isoform in mice (mNav1.x), rats (rNav1.x), and humans (hNav1.x). The canonical ABD is highlighted in red and canonical CK2 phosphorylation sites are highlighted in green. (B) Total sodium channel conductance in cultured eDRG neurons (WT; n = 9, WT + TBB; n = 9). (C) Total sodium channel conductance in cultured eDRG neurons pre-incubated with 250 μM 8-Br-cAMP (WT; n = 11, WT + TBB; n = 11). Statistical significance determined using a two-tailed unpaired student's t-test. All data presented as mean ± s.e.m.

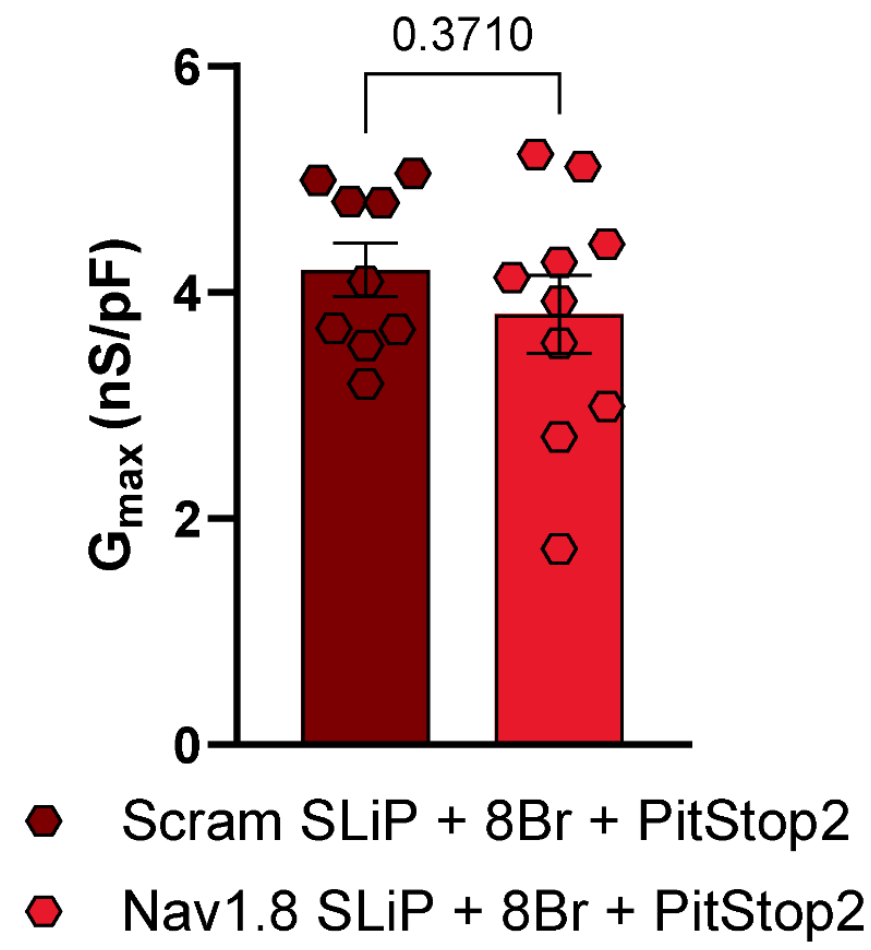

Figure S10. Inhibition of endocytosis reduces the efficacy of the Nav1.8 SLiP. Related to Figure 3 and 4. Total sodium channel conductance of eDRG neurons incubated with the clathrin-mediated endocytosis inhibitor Pitstop 2. Inhibiting clathrin-mediated endocytosis (CME) antagonized the cAMP kinase dependent effect of the Nav1.8 SLiP, partially rescuing the total sodium current density. Statistical significance determined using a two-tailed unpaired student's t-test. All data presented as mean ± s.e.m.

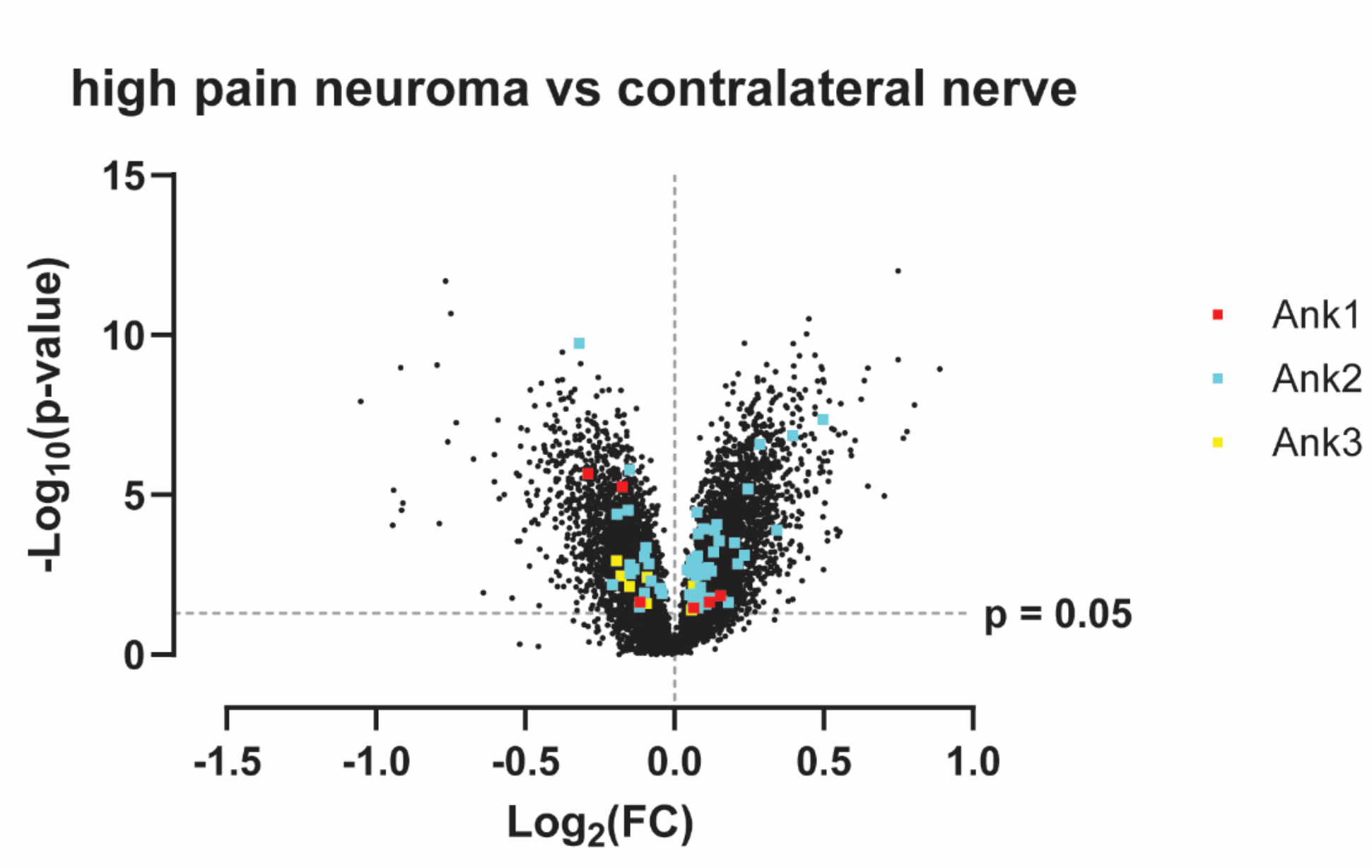

Figure S11. Phosphoproteomic analysis of peptides in neuroma and nerve reveals differential phosphorylation patterns for Ank1, Ank2, and Ank3. Related to Figure 6. Volcano-plot displaying phosphorylation patterns of proteins identified in high pain neuromas (pooled form 9 males and 9 females) compared to uninjured sciatic nerves.
